# Growth-Promoting Gold Nanoparticles Decrease Stress Responses in Arabidopsis Seedlings

**DOI:** 10.3390/nano11123161

**Published:** 2021-11-23

**Authors:** Eleonora Ferrari, Francesco Barbero, Marti Busquets-Fité, Mirita Franz-Wachtel, Heinz-R. Köhler, Victor Puntes, Birgit Kemmerling

**Affiliations:** 1 ZMBP, University of Tübingen, 72076 Tübingen, Germany; eleonora.ferrari@uni-tuebingen.de; 2 Catalan Institute of Nanoscience and Nanotechnology (ICN2), CSIC and BIST, Campus UAB, Bellaterra, 08193 Barcelona, Spain; fra.barbero@gmail.com (F.B.); victor.puntes@icn.cat (V.P.); 3 Universitat Autònoma de Barcelona (UAB), Bellaterra, 08193 Barcelona, Spain; 4 Applied Nanoparticles, S.L., 08018 Barcelona, Spain; marti.busquets@appliednanoparticles.eu; 5 Proteome Center, University of Tübingen, 72076 Tübingen, Germany; mirita.franz-wachtel@uni-tuebingen.de; 6 Animal Physiological Ecology, University of Tübingen, 72076 Tübingen, Germany; heinz-r.koehler@uni-tuebingen.de; 7 Institució Catalana de Recerca i Estudis Avançats (ICREA), 08010 Barcelona, Spain; 8 Vall d’Hebron Institut de Recerca (VHIR), 08032 Barcelona, Spain

**Keywords:** engineered nanomaterial (ENM), nanoparticle (NP), gold nanoparticle (AuNP), plant, *Arabidopsis thaliana*, plant growth, stress response, transcriptomics, proteomics

## Abstract

The global economic success of man-made nanoscale materials has led to a higher production rate and diversification of emission sources in the environment. For these reasons, novel nanosafety approaches to assess the environmental impact of engineered nanomaterials are required. While studying the potential toxicity of metal nanoparticles (NPs), we realized that gold nanoparticles (AuNPs) have a growth-promoting rather than a stress-inducing effect. In this study we established stable short- and long-term exposition systems for testing plant responses to NPs. Exposure of plants to moderate concentrations of AuNPs resulted in enhanced growth of the plants with longer primary roots, more and longer lateral roots and increased rosette diameter, and reduced oxidative stress responses elicited by the immune-stimulatory PAMP flg22. Our data did not reveal any detrimental effects of AuNPs on plants but clearly showed positive effects on growth, presumably by their protective influence on oxidative stress responses. Differential transcriptomics and proteomics analyses revealed that oxidative stress responses are downregulated whereas growth-promoting genes/proteins are upregulated. These omics datasets after AuNP exposure can now be exploited to study the underlying molecular mechanisms of AuNP-induced growth-promotion.

## 1. Introduction

Engineered nanomaterials (ENMs) are distributed into the environment in drastically increasing amounts, yet knowledge on the resulting effects of ENMs on the environment is limited [1,2]. Although naturally occurring nanomaterials have always existed, in the last decade the emission rate of anthropogenic nanoparticles (NPs), intentionally or unintentionally released, has been continuously rising [3,4]. For these reasons, novel nanosafety approaches to assess the environmental impact of ENMs are required [5].

Gold ENMs are used worldwide in various fields, including medicine, biology, chemistry, physics, electronics, and cosmetics [6,7,8]. The unique optical and electrochemical properties of AuNPs [9], as well as their accessibility for various surface functionalizations [10], have been exploited in many applications ranging from diagnostics and cancer therapy [11] to industrial catalysis [12] and water purification [13]. Furthermore, the latest developments in nanotechnology have opened up new opportunities in the food safety industry [14,15] and agronomy [16]. Several studies have shown that biosynthesized AuNPs have larvicidal and nematicidal effects in crop cultivation without adversely affecting their growth and development [17,18], implicating the application of more agro-ecological practices in the future.

The increasing production, use, and disposal of ENMs has translated into a higher and uncontrolled release of such materials in the environment [19]. As a result of the unique size-dependent physicochemical properties of NMs, such as higher reactivity than their respective bulk materials, concerns about potential adverse effects have arisen [20,21]. In order to assess the risk of ENMs on biosystems, a comprehensive characterization of the material is required, as their physicochemical properties and behavior after release into the environment depend on their chemical composition, size, shape, and surface [22,23]. Stable colloidal ENMs can be obtained by steric or electrostatic surface functionalization with coating materials [24,25]. Despite the stabilizing function of such surface modifications, it must be taken into account that the chemistry of coatings can change under natural conditions, affecting the properties of ENMs and their biocompatibility [26,27]. 

To study the risk of ENMs, ecological effects should be studied under natural conditions, but model systems also need to be optimized for stable, non-toxic assay conditions and adequate characterization of ENMs. As ENMs can change their properties in different environments, characterization is necessary for the starting material as well as under experimental conditions. 

Positive, negative, and no effects of ENMs on human health, animals, and plants have been described. In vitro toxicity studies of AuNPs did not find detectable changes in the concentration of inflammatory markers in either humans or animals [28,29,30,31]. On the other hand, AuNPs were associated with dose-dependent imbalances of oxidative stress levels in vitro [32,33], with higher doses of particles responsible for initial oxidative cell damage [34,35]. Many studies have reported that the effects of AuNPs on living organisms are strictly related not only to their concentration, but especially to their size and the physicochemical properties of the coatings [36,37]. Environmental AuNP concentration predicted by screening models are 0.14 μg L^−1^ in natural waters and 5.99 µg kg^−1^ in soils [38]. Under lab conditions, for these concentrations no measurable physiological effect on plants have been reported [39]. To evaluate whether AuNPs have any effect on plants, higher concentrations are tested to assess the maximum potential risk that could be caused by AuNP accumulation. The effects of AuNPs on plants were reviewed by Siddiqi and Husen [40]. Overall, despite a few contradictory studies, AuNPs have been found to have detrimental effects at high concentrations (≥100 mg/L) [41], with particles with a diameter below 5 nm showing increasing toxicity [42,43,44]. By contrast, lower concentrations of AuNPs can enhance seed germination and chlorophyll content, and improve growth and productivity in several crops and model plants under laboratory conditions [45,46,47]. Enhanced and reduced toxic effects and stress responses, such as reactive oxygen species (ROS) production and activation of immune responses, have both been reported in plants after AuNP treatment [40,47,48,49,50]. Furthermore, growth-promoting effects are common for nanofertilizers such as ZnNPs, for example, but were also observed for inert AuNPs [18,47]. AuNPs are widely inert and the special physicochemical properties of NPs can be studied without physiological side effects of the bulk material [51]. The mechanism underlying AuNP-induced growth-promotion is not yet understood. Understanding how NPs influence plant growth could be useful for improving crop yield in the future [52].

AuNP uptake is controversially discussed in the community, with studies showing uptake and even transport within the plant and others ruling it out [53,54,55,56]. The pore size of cell walls has been determined to be approximately 3–6 nm, a size that precludes the uptake of larger molecules, but some properties of NPs, such as surface charge, may induce morphological changes in the cell wall, thereby affecting pore size and uptake rate [57,58]. Though many plants have been tested for their ability to take up various kinds of ENMs, our knowledge is still limited and many aspects remain elusive. The mechanisms underlying the physiological effects caused by AuNPs in or outside the plant cells will be interesting to elucidate in the future.

Transcriptomics and proteomics studies after exposure to multi-walled carbon nanotubes (MWCNT), titanium dioxide (TiO_2_), cerium dioxide (CeO_2_), and silver (Ag) NPs have been reported in Arabidopsis plants [59,60,61,62]. Furthermore, Simon et al. [63] performed a transcriptome sequencing study on the eukaryotic green alga *Chlamydomonas reinhardtii* after treatment with Ag, TiO_2_, zinc oxide (ZnO) NPs, and quantum dots (QDs). Conversely, the transcriptome and proteome changes in plants exposed to AuNPs have not yet been adequately studied.

Here, we show that well-characterized AuNPs stabilized with sodium citrate and tannic acid (SCTA) are stable, sterilizable, and functional in promoting the growth of Arabidopsis seedlings and do not show any negative effects at moderate concentration levels. Stress responses are downregulated after AuNP exposure at the ROS burst level and also on the transcriptome and proteome level. We studied transcriptome and proteome changes after AuNP-SCTA treatment, and those omics data revealed candidate genes/proteins that could explain the growth-promoting effect of AuNPs on a molecular level.

## 2. Materials and Methods

### 2.1. AuNP Synthesis

Aqueous dispersions of citrate-stabilized AuNPs were synthesized following two kinetically controlled seeded growth approaches as reported by Bastús et al. [64] and Piella et al. [65]. Tetrachloroauric (III) acid trihydrate (99.9% purity), sodium citrate tribasic dihydrate (99%), and tannic acid were purchased from Sigma-Aldrich (Madrid, Spain). Briefly, 150 mL of sodium citrate (SC) aqueous solution (2.2 mM) were brought to a boil in a three-neck flask under reflux; subsequently, 1 mL of 25 mM chloroauric acid (HAuCl_4_) was injected into the citrate solution. After few minutes the solution became reddish, indicative of AuNP formation (~10 nm, seeds). Afterwards, different sequential steps of growth, consisting of sample dilution plus further addition of gold precursor, led to the desired AuNP size. In the second method, the main difference was the addition of traces of tannic acid (TA) (200 µM) as a co-reducer and an increase in the starting pH to produce highly monodispersed and stable AuNPs. Several batches of the AuNPs were synthesized with and without the addition of TA. All batches presented very similar features and produced similar results in the assays described. The AuNPs were synthesized by the Catalan Institute of Nanoscience and Nanotechnology and purchased from Applied Nanoparticles SL. 

The AuNP dispersions were concentrated by Amicon^®^ Ultra Centrifugal Filters (100 kDa) (Merck, Darmstadt, Germany).

### 2.2. AuNP Characterization

#### 2.2.1. Size Determination by Electron Microscopy

The diameter of the synthesized AuNPs was measured by analysis of images obtained by scanning electron microscopy (SEM) with FEI Magellan XHR SEM (FEI, Hillsboro, OR, USA) in transmission mode (STEM) operated at 20 kV. Samples were prepared by drop-casting 3 μL of the NP dispersion onto a carbon-coated copper TEM grid and left to dry under mild vacuum. To prevent aggregation of the NPs during the drying procedure, they were previously conjugated with 55 kDa polyvinylpyrrolidone (PVP) (Sigma-Aldrich, Madrid, Spain). More than 500 particles from different regions of the grid were measured.

#### 2.2.2. UV-Vis Spectroscopy

The UV-Vis absorption spectra of AuNPs are due to the collective oscillation of their metallic surface electrons, called localized surface plasmon resonance (LSPR). The LSPR profile and maximum position strictly depend on the material, shape, and size of the NPs, as well as the refractive index of the solvent and the vicinity of the NP surfaces. The LSPR profile is highly sensitive to NP aggregation. At inter-particle distances that are less than their diameter, the NP near-field electromagnetic coupling applies, leading to significant UV-Vis spectra changes that translate to a LSPR red-shift and/or to the occurrence of a second peak at a higher wavelength. For large aggregates, an increase in the baseline can be observed [66,67,68].

UV-Vis spectra were acquired with a Shimadzu UV-2400 spectrophotometer (SSI, Kyoto, Japan). One mL of sample was placed in a plastic cuvette and analyses were performed at time zero or over time in the 300–800 nm range at room temperature. In the case of solidified media, samples were poured into the cuvette prior to jellification. MilliQ water or ½ Murashige and Skoog (MS) agar (Duchefa, Haarlem, The Netherlands) were taken as reference for the different samples. 

#### 2.2.3. Size and Zeta Potential Measurements

Laser doppler velocimetry and dynamic light scattering were used to determine the Z potential and the hydrodynamic diameter of the AuNPs, respectively, employing a Malvern Zetasizer Nano ZS instrument (light source wavelength at 638.2 nm; detector at a fixed scattering angle of 173°) (Malvern Panalytical Ltd., Malvern, UK). Measurements were performed at 25 °C. Diameters were reported as Z-average and polydispersity index (PDI) calculated by cumulative analysis. 

#### 2.2.4. Au Quantification

Samples were digested with aqua regia (1:3 HNO_3_ (70%):HCl (37%)) for 24 h and then diluted with MilliQ water to be further analyzed by induced coupled plasma-mass spectroscopy (ICP-MS) using an ICP-MS NexION 300 from Perkin Elmer (Shelton, CT, USA).

### 2.3. AuNP Sterilization

AuNP suspensions were sterilized by filter sterilization with cellulose mixed ester (CME) and polyethersulfone (PES) filters (Carl Roth, Karlsruhe, Germany), both with a pore size of 0.2 μm, according to the manufacturer’s protocol. UV-Vis spectra of AuNPs before and after filtration were acquired as previously mentioned. 

### 2.4. Plant Growth Conditions and Plant NP Exposition

#### 2.4.1. Seed Sterilization

*Arabidopsis thaliana* ecotype Columbia 0 (Col-0) seeds were surface sterilized by chlorine gas treatment in a desiccator containing 50 mL of 12% sodium hypochlorite and 2 mL of 37% HCl for 4 h. The chemicals were purchased from Merck (Darmstadt, Germany). The seeds were dried in a laminar air flow sterile bench for 30 min. The seeds were grown on agar-solidified medium or in hydroponic cultures.

#### 2.4.2. Plant Growth Conditions

All plants were grown in long day conditions with 16 h light, 8 h dark, 22 °C, 110 μmol m^−2^ s^−1^ light, and 60% relative humidity.

#### 2.4.3. NP Exposure in Agar-Solidified Medium

Sterilized seeds were sown on agar plates containing ½ MS plant medium and stratified at 4 °C for 2 days in the dark. Afterwards, plates were incubated for 7 days under controlled long day conditions. Plates were placed vertically to allow root growth along the agar surface. The reducing and stabilizing agents sodium citrate and tannic acid (SCTA) or AuNP-SCTA were mixed with the medium at the indicated concentrations before jellification. Physicochemical characterization of AuNP-SCTA dispersed in ½ MS agar showed high colloidal stability up to 3 weeks, allowing for 1 week exposure experiments.

#### 2.4.4. NP Exposure in Hydroponic Culture

Sterilized seeds were sown on a thin layer of ½ MS agar medium and stratified at 4 °C for 2 days in the dark. Subsequently, the seeds were germinated and grown for 2 weeks under controlled long day conditions. The seedling roots grew through the agar into liquid ½ MS plant medium. After 2 weeks, SCTA or AuNP-SCTA were mixed in the indicated concentration with the ½ MS medium and were incubated for 6 h. Since UV-Vis spectroscopic analyses of AuNP-SCTA dispersed in ½ MS revealed no changes in the shape of the spectrum at 6 h, whereas a typical aggregation profile was shown after 9 h, this interval was chosen for short-term experiments.

### 2.5. Physiological Effects

*Arabidopsis thaliana* seedlings were grown in agar-solidified medium, as described previously, with SCTA or AuNP-SCTA to a final concentration of 10 mg/L. Photographs of 7 day-old seedlings were taken. Growth parameters, i.e., rosette diameter, primary root length, and lateral root length, were measured using the software ImageJ. The lateral root number was determined by counting the number of lateral roots per seedling with 20 seedlings being analyzed for each individual parameter (n = 20).

### 2.6. Statistical Analysis

Statistical significance between groups was evaluated using one-way ANOVA combined with Tukey’s honest significant difference (HSD) test. FOX assay data were tested with a two-way nested ANOVA followed by Dunnett’s post-hoc test; data were normally distributed (Shapiro–Wilk test) and showed homogeneity of variances (Levene’s test). Significant differences are indicated with different letters (*p* < 0.01). Statistical evaluations were performed using JMP (version 15.0.0, Heidelberg, Germany) software.

### 2.7. Detection of Immune-Related Responses

#### 2.7.1. Oxidative Burst

Production of reactive oxygen species (ROS) was measured in a luminol-based assay using a microplate luminometer (CentroPRO LB 962; Berthold Technologies, Bad Wildbad, Germany) as described by Albert et al. [69]. The elicitor flg22 (final concentration 100 nM) was used in the assay as positive control. The horseradish peroxidase, in the presence of ROS, catalyzed the oxidation of luminol to 3-aminophthalate with emission of light at 428 nm. The monitored oxidative burst was measured as emitted light and recorded as relative light units (RLU). The ROS burst was monitored for 30 min for 3 plants per treatment and three leaf pieces per plant (n = 9).

#### 2.7.2. FOX Assay

The level of lipid hydroperoxides (LOOHs) was assessed with the modified colorimetric ferrous oxidation xylenol orange (FOX) assay as described by Hermes-Lima et al. [70] and adjusted by Schmieg et al. [71]. Leaves of 5 week-old *A. thaliana* plants were cut into square pieces (about 2 mm^2^) and left to equilibrate overnight in milliQ water. Then the leaf pieces were elicited for 30 min with flg22 (100 nM) or the tested compounds and immediately stored at −80 °C. Three plants and three leaf pieces per plant (n = 9) were used for each sample. Samples were homogenized in ice-cold HPLC-grade methanol in a 1:15 ratio, and 30 μL of the supernatant was used in the reaction mixture. Cumene hydroperoxide equivalents (CHPequiv./mg wet weight) were calculated using the following equation:

$$CHPE=\frac{ABS570}{\mathrm{ABS}570+\mathrm{CHP}}\times Volume\;CHP\times \frac{Total\;Volume}{Sample\;Volume}\times Dilution\;Factor\;=\frac{ABS570}{ABS570+CHP}\times 1\times \frac{200}{30}\times 15$$

### 2.8. Transciptomics

#### 2.8.1. RNA Extraction

RNA was extracted from 100 mg of Arabidopsis seedling roots treated for 6 h and 7 d with 10 mg/L Au-SCTA or SCTA (SC 2.2 mM; TA 200 µM) in triplicate using the RNeasy Plant Mini Kit (QIAGEN, Hilden, Germany) followed by on-column DNA digestion with the RNase-Free DNase Set (QIAGEN, Hilden, Germany). The total RNA concentration, RNA Integrity Number (RIN) value, and rRNA ratio (28S/18S) were evaluated using Agilent2100 Bionalyzer (RNA 6000 Nano Kit; Agilent, Waldbronn, Germany).

#### 2.8.2. Transcriptome Sequencing Analysis

The BGI Group (Shenzhen, China) performed the total transcriptome sequencing (RNA-Seq) analysis. Samples were sequenced on the Illumina HiSeq platform. The internal software SOAPnuke v1.5.2 was used to filter low-quality reads, reads with adaptors, or those containing more than 5% of unknown bases (N). Genome mapping of clean reads was performed using HISAT v2.0.4 (Hierarchical Indexing for Spliced Alignment of Transcripts) software [72]. The assembler of RNA-Seq alignments into potential transcripts StringTie v1.0.4 was used to reconstruct transcripts [73]. Cuffcompare, a tool of Cufflinks [74], was used to identify novel transcripts by comparing reconstructed transcripts with genome reference annotation information. The coding ability of those new transcripts was predicted using CPC v0.9-r2 [75]. After novel transcript detection, novel coding transcripts were merged with reference transcripts to get a complete reference and clean reads were mapped to it using Bowtie2 v2.2.5 [76]. For each sample, the gene expression level was calculated with RSEM, a software package for estimating gene and isoform expression levels from RNA-Seq data [77]. Differentially expressed genes (DEGs) were detected with the nonparametric approach NOIseq method (parameters: fold change ≥ 2.00 and probability ≥ 0.8) as described by Tarazona et al. [78].

### 2.9. Mass Spectrometry Analysis

#### 2.9.1. Total Protein Extraction 

For total protein extraction from seedlings, 100 mg of material were ground in liquid nitrogen and mixed in a ratio of 1:3 with ice-cold extraction buffer (10% glycerol, 150 mM Tris/HCl, pH 7.5, 1 mM EDTA, 150 mM NaCl, 10 mM DTT, 0.2% Nonidet P-40, 2% PVPP, 1 tablet of proteinase inhibitor cocktail (Roche, Mannheim, Germany) per 10 mL solution). Protein extraction was performed on a rotor at 4 °C for 1 h and the extract was purified by a centrifuging at 4 °C, 5000× *g* for 20 min. The supernatant was then transferred through a one-layer Miracloth (Merck, Darmstadt, Germany) in a fresh pre-chilled 1.5 mL tube on ice. 

#### 2.9.2. NanoLC-MS/MS Analysis

The Proteome Center Tübingen performed the nanoscale liquid chromatography coupled to tandem mass spectrometry (LC-MS/MS) on total protein extracts as described. 

Proteins were purified in a 12% NUPAGE Novex Bis-Tris Gel (Invitrogen, Karlsruhe, Germany) for 10 min at 200 V and stained with Colloidal Blue Staining Kit (Invitrogen, Karlsruhe, Germany). In-gel digestion of proteins was performed as previously described [79]. Extracted peptides were first desalted and then labeled using C18 StageTips [80] as described elsewhere [81]. Samples were labeled with dimethyl “light” ((CH_3_)_2_) and dimethyl “intermediate” ((CH_1_D_2_)_2_). Complete incorporation levels of the dimethyl labels were achieved in all cases.

Eluted peptides were mixed in a 1:1 ratio according to the measured protein amounts. The analysis of the peptide mixture was performed on an Easy-nLC 1200 system coupled to an LTQ Orbitrap Elite or a QExactive HF mass spectrometer (all Thermo Fisher Scientific) as described elsewhere [82] with slight modifications: Peptides were injected onto the column in HPLC solvent A (0.1% formic acid) at a flow rate of 500 nL/min and subsequently eluted with a 227 min (Orbitrap Elite) or 127 min (QExactive HF) gradient of 10–33–50–90% HPLC solvent B (80% ACN in 0.1% formic acid). During peptide elution the flow rate was kept constant at 200 nL/min. 

In each scan cycle, the 15 (Orbitrap Elite) or 12 (Q Exactive HF) most intense precursor ions were sequentially fragmented using collision-induced dissociation (CID) and higher energy collisional dissociation (HCD) fragmentation, respectively. In all measurements, sequenced precursor masses were excluded from further selection for 60 (Orbitrap Elite) or 30 s (Q Exactive HF). The target values for MS/MS fragmentation were 5000 and 10^5^ charges, and for the MS scan 10^6^ and 3 × 10^6^ charges. 

#### 2.9.3. MS Data Processing

The MS data were processed with MaxQuant software suite v1.5.2.8 and v1.6.3.4 (Cox and Mann 2008), respectively. A database search was performed using the Andromeda search engine [83], which is a module of the MaxQuant. MS/MS spectra were searched against an *Arabidopsis thaliana* database obtained from Uniprot, and a database consisting of 285 commonly observed contaminants. In the database search, full tryptic specificity was required and up to two missed cleavages were allowed. Protein N-terminal acetylation and oxidation of methionine were set as variable modifications. Initial precursor mass tolerance was set to 4.5 ppm and to 0.5 Da at the MS/MS level (CID fragmentation), or 20 ppm (HCD fragmentation). Peptide, protein, and modification site identifications were filtered using a target-decoy approach at a false discovery rate (FDR) set to 0.01 [84]. For protein group quantitation a minimum of two quantified peptides were required. 

Perseus software (v1.6.1.3), a module from the MaxQuant suite [85], was used for calculation of the significance B (p_sigB_) for each protein ratio with respect to the distance of the median of the distribution of all protein ratios as well as the intensities. All proteins with a fold change ≥2.00- and p_sigB_ <0.01 in a pairwise comparison were considered to be differentially expressed.

## 3. Results

### 3.1. Physicochemical Characterization of AuNPs Dispersed in Plant Growth Media

**Figure 1:**
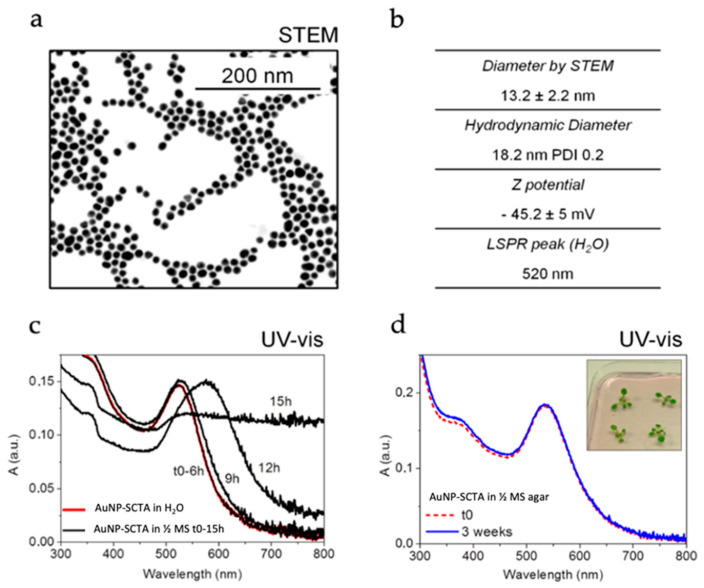
Physicochemical characterization of AuNP-SCTA dispersed in H_2_O, ½ MS, and ½ MS agar. (**a**) Bright field—scanning transmission electron microscopy (STEM) of AuNP-SCTA. (**b**) NP average diameter measured by STEM; hydrodynamic diameters (in H_2_O) measured by dynamic light scattering, reported as Z average and poly dispersity index (PDI); Z potential of the AuNP-SCTA dispersed in H_2_O (pH 6.5, conductivity 0.85 mS/cm). (**c**) UV-Vis spectra of AuNP-SCTA dispersed in H_2_O (red) and over time (from time 0 to 15 h) in ½ MS (black). (**d**) UV-Vis spectra of AuNP-SCTA dispersed in ½ MS agar at time 0 (red dashed) and after 3 weeks of exposure (blue); representative photograph of Arabidopsis seedlings germinated and grown for 1 week on an agar plate containing ½ MS and AuNPs, showing a typical reddish color of non-aggregated AuNPs-SCTA. Absorbance A in arbitrary units (a.u.). All experiments were repeated twice with similar results.

Two different types of gold nanoparticles (AuNPs) with an average diameter of about 12 nm were synthesized with the two seeded-growth methods reported by Bastús et al. [64] and Piella et al. [65], with the only difference being the addition of tannic acid (TA), which can interact with the NP surface, inferring higher colloidal stability. The physicochemical characterization of one of the used batches of AuNPs prepared in the presence of TA (AuNP-SCTA) is shown in Figure 1a,b, whereas the characterization of AuNP-SC is reported in Supplemental Figure S1a,b.

It is important to characterize the evolution of the AuNPs once dispersed in the working media to correctly correlate the pristine and the evolving NP features with the observed biological effects [86]. Thus, over time, physicochemical characterization of AuNP-SC and AuNP-SCTA in the used working media, i.e., ½ MS and agar-solidified ½ MS media, was performed. Once dispersed in ½ MS, AuNP-SC underwent fast aggregation, pointed out by an immediate emergence of a second localized surface plasmon resonance (LSPR) peak at around 650 nm in the UV-Vis spectra (Supplemental Figure S1c) [87]. This aggregation was probably due to the increase in the ionic strength by mono- and divalent inorganic ions in the media (½ MS has a salinity of 23 mM). The ions in the media can screen the negative charges provided by the SC present on the surface of the AuNPs, responsible for the electrostatic repulsion between particles [88,89].

By contrast, the UV-Vis spectra of AuNP-SCTA dispersed in ½ MS showed no changes until 6 h of exposure. After 9 h, changes in the spectrum shape were observed, showing the start of a typical aggregation profile that led to complete aggregation after 15 h, detectable by a drastic change in the UV-Vis spectrum (Figure 1c) [87]. This result indicates that AuNP-SCTA had a good colloidal stability up to 6 h of exposure to the hydroponic medium, whereas after 9 h the NPs started to slowly aggregate. Therefore, in this study all experiments carried out in hydroponic cultures were short time exposures, in a time range of 6 h. The presence of TA was the only difference between the two types of AuNPs. Thus, we hypothesize that this organic molecule functions as a NP stabilizer, increasing the particle stability against salt-driven aggregation. TA confers an effective higher surface charge or partial steric stabilization, preventing NP aggregation. Regarding the observed aggregation of the AuNP-SCTA in ½ MS after 9 h, it could be speculated that organic molecules (e.g., sucrose), present in the medium in excess compared to the NP stabilizers, could progressively replace the NP stabilizers on the NP surface, conferring a negative effect on stabilization and supporting aggregation. However, further studies will be necessary to precisely understand the role and nature of TA in the stabilization of AuNP-SCTA. 

UV-Vis spectroscopy of AuNP-SCTA exposed to ½ MS agar showed high stability at least up to 3 weeks (Figure 1d), allowing for long-term exposure experiments. Conversely, AuNP-SC dispersed in ½ MS agar showed an initial aggregation that, unlike in ½ MS, did not evolve over time (Supplemental Figure S1d), probably due to the interaction with agar molecules and the fast viscosity increase due to medium jellification as well as to the reduced number of particles (aggregation is directly proportional to concentration). Note that below 10^10^ NP mL^−1^ the collision probability decreases to almost zero, so even if their surface is not passivated, NPs do not aggregate.

In light of these observations, the AuNP-SCTA were chosen for the following physiological and molecular studies, permitting all the experiments to be conducted with stable AuNPs, thereby allowing for the correct NP size to be correlated with the observed effects on Arabidopsis.

Several AuNP-SCTA batches with very similar physicochemical features were produced and tested. The results of the physiological studies after AuNP-SCTA treatment were fully reproducible between the different batches, showing that the synthesis protocol is very robust and produced reliable results.

### 3.2. AuNP-SCTA Sterilization 

**Figure 2:**
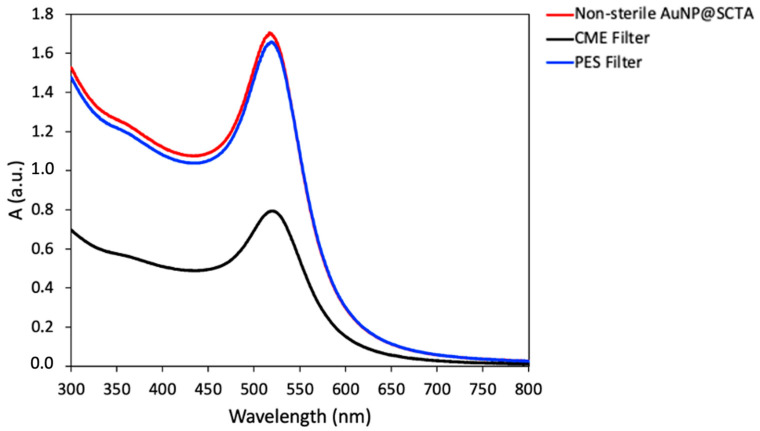
UV-Vis spectra of AuNP-SCTA before and after sterilization with different filter materials. UV-Vis spectra of AuNP-SCTA before (red dashed) and after sterilization with PES (blue) and CME (black) filters were acquired with a Shimadzu UV-2400 spectrophotometer. Absorbance A in arbitrary units (a.u.). The experiment was repeated twice with similar results.

In order to grow seedlings under sterile conditions and to discriminate the AuNP effects from the possible physiological and molecular changes induced in plants by microbial contaminants such as, e.g., (pathogenic) bacteria and fungi, the sterility of the colloidal solution is a fundamental requirement. To sterilize AuNP-SCTA solutions, physical filtration methods were chosen. Two different filter materials, i.e., cellulose mixed ester (CME) and polyethersulfone (PES), both with a pore size of 0.2 μm, were tested. To analyze possible changes in the particle concentration or their aggregation state, UV-Vis spectra before and after filtration were acquired (Figure 2). Both filtering procedures were effective in removing all contaminating microorganisms and allowed plant cultivation under sterile conditions. The CME filter, a standard hydrophilic membrane commonly used for a broad range of applications, was shown to significantly affect the amplitude of the spectra, revealing a drastically reduced AuNP-SCTA concentration. By contrast, the PES filter, a hydrophilic and low protein-binding membrane, did not change the spectra and therefore did not affect the concentration of AuNP-SCTA. No changes in the overall shape of the UV-Vis spectrum were observed, indicating that no alterations of the physicochemical properties of the AuNPs occurred. Therefore, PES membranes were used in all subsequent experiments for NP sterilization.

### 3.3. Physiological Effects of AuNPs

Although gold (Au) can be present in the environment from natural sources, in the last decade the increased use and disposal of AuNPs has affected the level of this chemical element in soil and water [1]. Although many studies on the accumulation and physiological effects of Au in various plant species have been conducted [90], a comprehensive investigation on AuNP fate and action after their release into plant growth media and their effects on plants at the physiological, transcriptomic, and proteomic level is missing. In the present study, *Arabidopsis thaliana* was used as a model plant to investigate the effects of AuNPs on growth and development. As shown in Supplemental Figure S2b, AuNP-SCTA in a range from 0 to 20 mg/L affected Arabidopsis root growth in a dose-dependent manner with a maximal effect at 10 mg/L, whereas the NP stabilizer SCTA (SC 2.2 mM; TA 200 µM) did not affect the primary root length at any of the tested concentrations (Supplemental Figure S2a). For this reason, 10 mg/L AuNP-SCTA was chosen as the final concentration in all subsequent experiments.

Physiological analyses were performed in order to evaluate Arabidopsis responses to abiotic stress caused by AuNP-SCTA exposure. Seedlings, grown under controlled long day conditions, were harvested after 7 days, and representative parameters were recorded, i.e., primary root length, rosette diameter, number of lateral roots, and lateral root length. For each parameter, another set of plants was grown in the presence of SCTA (SC 2.2 mM; TA 200 µM) as a control.

**Figure 3:**
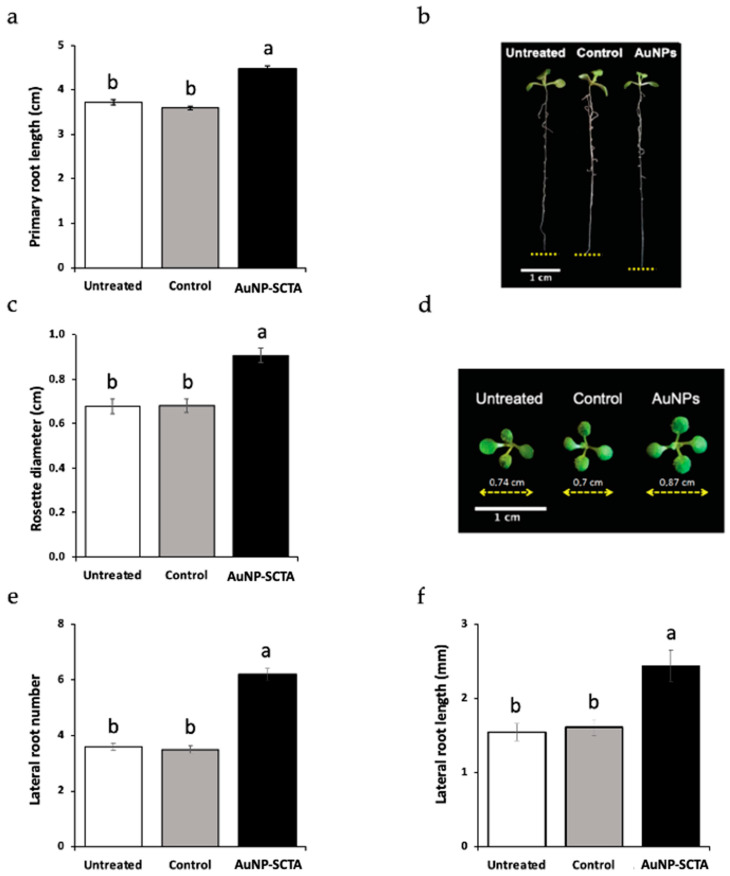
AuNP-SCTA enhances growth of Arabidopsis seedlings. Wild-type Arabidopsis seedlings were grown for 7 d on agar-solidified ½ MS medium containing 10 mg/L of AuNP-SCTA or SCTA (SC 2.2 mM; TA 200 µM) (control) or on un-supplemented medium (untreated). Growth parameters were scored: (**a**) primary root length and (**b**) representative picture of (**a**). (**c**) Rosette diameter and (**d**) representative photograph of (**c**). (**e**) Lateral root number. (**f**) Lateral root length. Results shown are means ± SE with n = 20. Different labels a and b indicate statistically different groups according to multiple comparisons following one-way ANOVA analysis at a probability level of *p* < 0.01. All experiments were repeated twice with similar results.

Although SCTA did not affect plant growth and development, AuNP-SCTA had a positive influence on all parameters tested. Seedlings germinated and grown on AuNP-SCTA-containing medium developed a longer primary root, with an enhancement of 1.2 folds compared to control seedlings (Figure 3a). Furthermore, the lateral root number and length were positively affected upon AuNP-SCTA treatment, displaying, compared to the controls, an increase of 1.7- and 1.5-fold, respectively (Figure 3e,f). Shoot development was also influenced by AuNP-SCTA exposure in the same way as the root system (Figure 3c). The size of the rosette diameters was enhanced by 1.3-fold in comparison to the control seedlings. These data show that AuNP-SCTA is not acutely toxic to plants, but rather have a positive effect on plant growth.

### 3.4. Immune Responses upon AuNP Treatment

NPs have been reported to affect the innate immune system in animals [5]. To assess whether they have an influence on plant immune responses, different innate immune defensive reactions in plants were evaluated. The production of reactive oxygen species (ROS) in the apoplast and lipid peroxidation are typical cellular events triggered by the plant surveillance system that detects highly conserved microbe- or pathogen-associated molecular patterns (M/PAMPs) via cell surface-located pattern-recognition receptors (PRRs) in a process called pattern-triggered immunity (PTI) [91,92].

**Figure 4:**
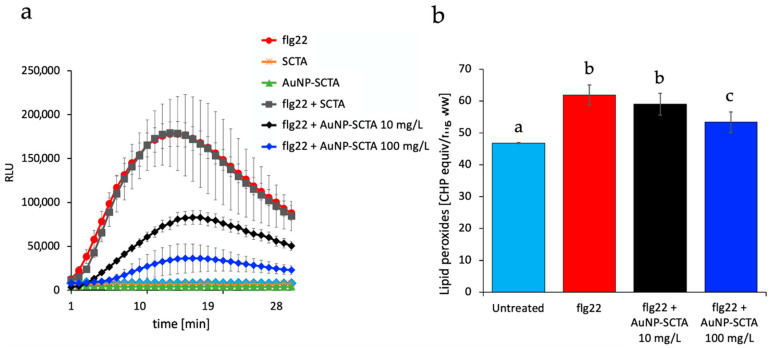
AuNP-SCTA decreases ROS production and lipid peroxidation levels. (**a**) ROS production measured with a luminol-based assay in leaf squares of Arabidopsis Col-0. ROS production is represented as relative light units (RLU) after elicitation with milliQ water (untreated control), flg22 (100 nM) (positive control), AuNPs-SCTA (100 mg/L), SCTA (control), flg22 + SCTA, or flg22 + AuNPs-SCTA 10 or 100 mg/L. Results are mean ± SE (n = 9). The experiment was repeated two times with similar results. (**b**) Lipid peroxides level, expressed as CHP equiv./mg ww, was measured in Arabidopsis leaves with the FOX assay. The results after treatment with milliQ water (untreated), flg22 (100 nM) (positive control), or flg22 + AuNPs-SCTA (10 or 100 mg/L) are presented as mean ± SE of three independent experiments. Based on two-way nested ANOVA followed by Dunnett’s post-hoc test, data were normally distributed (Shapiro–Wilk test) and showed homogeneity of variances (Levene’s test). Different letters indicate statistically significant differences at *p* < 0.01. Different labels a–c indicate statistically different groups according to multiple comparisons following two-way nested ANOVA analysis at a probability level of *p* < 0.01.

The cellular response of *Arabidopsis thaliana* to abiotic stress resulting from NP exposure was initially measured as reactive oxygen species (ROS) production or oxidative burst, using a luminol-based chemiluminescence assay. As shown in Figure 4a, no ROS production was detected after exposure to milliQ water (untreated control), AuNP-SCTA (100 mg/L), or coating solution SCTA (SC 2.2 mM; TA 200 µM) (control). This also confirms that the NP suspensions were free of endotoxins such as LPS that would induce ROS in plants [93]. As positive control, the PAMP flg22 was added at a final concentration of 100 nM. The same concentration of the elicitor was used as treatment also in combination with AuNP-SCTA (10 or 100 mg/L) or SCTA as control. Although the coating solution did not affect the level of ROS production caused by flg22 treatment, AuNP-SCTA influenced the level of recorded ROS. In particular, in the presence of 10 mg/L AuNP-SCTA the PAMP (flg22) signal decreased. Furthermore, a 10× higher NP concentration (100 mg/L) was tested, and a further decrease in the level of ROS was detected. 

In order to discriminate between a real decrease in the ROS production and a mere technical interference with the light detection, a lipid peroxidation assay was performed. Cellular and organelle membranes, due to their high polyunsaturated fatty acid (PUFA) content, are particularly susceptible to ROS-induced peroxidation [94]. The applied colorimetric ferrous oxidation xylenol orange (FOX) assay was modified to quantify lipid hydroperoxides (LOOHs) in plant extracts. Upon treatment with 10 and 100 mg/L AuNP-SCTA plus flg22, the level of lipid peroxidation decreased significantly in comparison to flg22 alone (Figure 4b). Treatment with 100 mg/L AuNP-SCTA resulted in a more pronounced decrease in the lipid hydroperoxide level compared to 10 mg/L AuNP-SCTA. The FOX assay confirmed the oxidative burst assay results, clearly pointing out that co-exposure to PAMPs and AuNP-SCTA reduced the PAMP-induced ROS burst and subsequent lipid peroxidation. The underlying mechanism of this effect is still elusive, but the data suggest that AuNP-SCTA might be able to detoxify ROS and shift the balance between growth and immunity trade-off to the growth side.

### 3.5. Transcriptomics Analysis of AuNP-SCTA-Exposed Arabidopsis Seedlings

To untie the molecular nature of the plant–AuNP interaction, whole transcriptome analyses were performed on Arabidopsis seedling roots after short (6 h) and long (7 d) exposure to 10 mg/L AuNP-SCTA in hydroponic culture and agar-solidified medium (6 h and 7 d, respectively). As controls, the seedlings were treated with SCTA (2.2 mM SC; 200 µM TA). Samples were sequenced with an Illumina HiSeq platform. The average genome mapping rate was 94.66% and the average gene mapping rate was 92.04%. Raw data for both experimental conditions and all three replicates are shown in Supplemental Table S1. 

**Figure 5:**
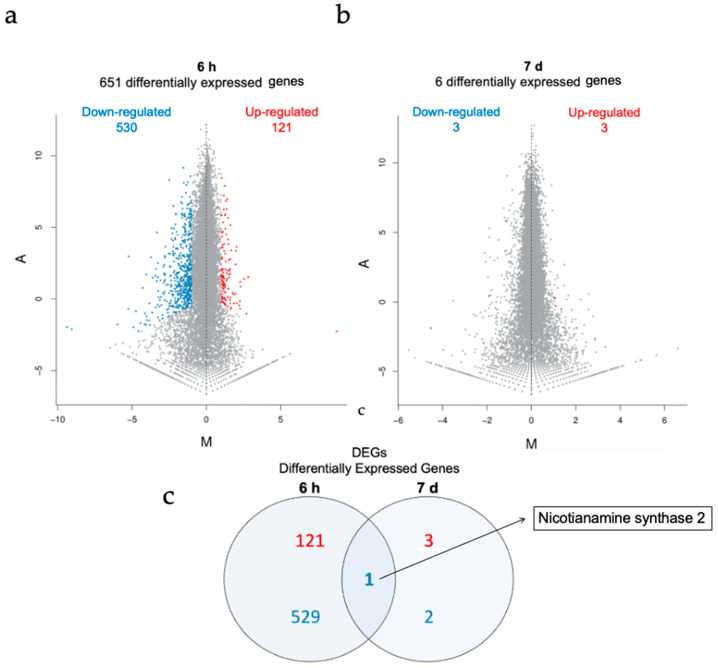
DEGs after AuNP-SCTA treatment. MA plot representing DEGs (upregulated genes: red dots; downregulated genes: blue dots) and non-DEGs (grey dots) in Arabidopsis seedling roots after (**a**) short- and (**b**) long-term AuNP-SCTA treatment (6 h and 7 d, respectively), detected by RNA-seq data analysis. The X-axis represents value M (log2 transformed fold change of a gene expression value) and the Y-axis represents value A (log2 transformed mean expression level). (**c**) Venn diagram displaying the total number of up- (red) and downregulated (blue) differentially expressed genes in both treatments and the name of the single overlapping gene.

As shown in Figure 5, a total of 651 differentially expressed genes (DEG) were identified after short-term treatment and 6 DEGs after long-term exposure. Whereas 121 genes were upregulated after 6 h of AuNP treatment, 530 genes were downregulated. After 7 d, 3 genes were upregulated and 3 genes were downregulated. DEGs with expression information are listed in Supplemental Table S2. Gene ontology (GO) (molecular biological function, cellular component, and biological process) and Kyoto Encyclopedia of Genes and Genomes (KEGG) pathway classification are reported in Supplemental Figures S3 and S4. In both conditions, genes involved in the response to external stimuli and cellular and metabolic processes are overrepresented within the DEGs. In particular, after short-term exposure the majority of genes involved in disease resistance, defense response, response to oxidative stress, and metal response were downregulated. This indicates that immune and oxidative stress responses were negatively affected during AuNP exposure. 

DUF642 L-GalL-responsive gene 2 (DGR2, At5g25460), a gene involved in growth and development of Arabidopisis plants, was up-regulated. DGR2 has a key role in Arabidopsis root elongation and shoot development [95,96]. Downregulation of immune response genes and upregulation of growth factors indicate a shift in the trade-off between immune and growth effects and may explain the growth-promoting effects of AuNPs. The Nicotianamine synthase 2 gene, (NAS2, At5g56080), the only shared DEG between the two conditions, encodes for a protein involved in the synthesis of nicotianamin. Mutants in NAS2 show altered metal contents, indicating a role in metal uptake or response [97] (Supplemental Table S2). After 7 d of NP exposure, only 6 DEGs were detected, compared to the 651 genes identified after 6 h, clearly pointing out that transcriptome changes are relevant only at early time points after AuNP treatment.

### 3.6. Proteomic Analysis of the Effect of AuNP-SCTA in Arabidopsis

To further understand the mechanisms underlying the effects of AuNPs on *Arabidopsis thaliana* seedlings, proteomic analyses were performed on seedlings using mass spectrometry. Global changes in protein expression were investigated in Arabidopsis seedlings in the same experimental setup as used for the transcriptome analyses. Protein extracts were analyzed via nano-liquid chromatography double mass spectrometry (NanoLC-MS/MS-spectrometry).

**Figure 6:**
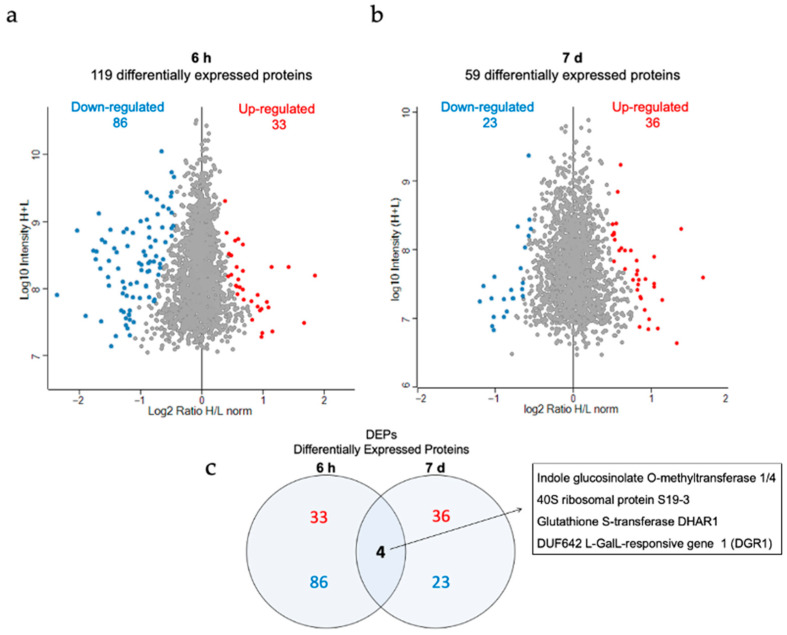
DEPs after AuNP-SCTA treatment. Volcano plots representing DEPs (upregulated proteins: red dots; downregulated proteins: blue dots) and non-DEPs (grey dots) in Arabidopsis seedlings after (**a**) short- and (**b**) long-term AuNP-SCTA treatment (6 h and 7 d, respectively) and detected by nano-liquid chromatography with tandem mass spectrometry (NanoLC-MS/MS) of total protein extracts. The X-axis represents the log_2_ transformed fold changes; the Y-axis represents the log_10_ transformed intensity (in log10), with H representing the treatment and L the control. (**c**) Venn diagram displaying the total number of up- (red) and downregulated (blue) differentially expressed proteins in both treatments and the protein names of the 4 overlapping proteins.

As shown in Figure 6, from a total of 2727 detected proteins after 6 h exposure and 2503 after 7 d exposure, 119 and 59 differentially expressed proteins (DEPs), respectively, were identified. All identified up- and downregulated proteins, along with their expression profiles, are listed in Supplemental Table S3. Furthermore, we sorted the DEPs into gene ontology (GO) categories (molecular biological function, cellular component, and biological process) and KEGG pathways, as shown in Supplemental Figures S5 and S6. DEPs significantly overrepresented after both treatments were involved in metabolic processes, protein synthesis, and response to stimuli (Supplemental Table S3). 

Oxidative stress-related proteins were mainly downregulated, as shown on the transcriptome level. The overlap analysis of the different timepoints revealed the protein DGR1 (DUF642 L-GalL-responsive gene 1, At1g80240), which was investigated for its role during the development of *Arabidopsis thaliana* [95]. After 7 d of treatment DGR1 and DGR2 were both upregulated, whereas after 6 h of treatment only DGR1 was initially downregulated. In the transcriptome analyses, the gene encoding for DGR2 was also detected to be upregulated. GSTF6 (Glutathione S-transferase F6, At1g02930), another DEG shared between treatments, encoded for a downregulated glutathione transferase involved in defense mechanisms (Supplemental Table S4). The finding of DGR2 and GSTF6 in both DEGs and DEPs indicates that these were reproducibly and robustly regulated genes/proteins upon AuNP exposure. As DGR1 and DGR2 have been previously described to be involved in growth and development, the differential regulation of these genes/proteins may explain why AuNPs have a positive effect on Arabidopsis growth. Our well-controlled transcriptome and proteome dataset provides a source for future analysis of the molecular mechanism underlying AuNP-induced growth-promotion.

## 4. Discussion

The widespread use of NP-containing products has led to the direct exposure of the terrestrial environment to these nanosized materials, raising concerns regarding their safety and biocompatibility with both living organisms and the environment [1,16,98,99,100]. Therefore, the risks and hazard assessment of NP exposure for plants, soil organisms, and consequently, humans, as a result of contamination of the food chain need to be addressed [1,101,102]. To date, despite recent developments in plant nanotoxicology, an unequivocal understanding of the effects of NPs on terrestrial plants is lacking, with fundamental information gaps about their mechanisms of action [103,104,105].

Plant-based nanosafety research focuses on a number of key aspects, i.e., the physicochemical properties of NPs such as material, size, and surface chemistry; the interaction of NPs with the surrounding environment; and the plant type and route of exposure [104,105,106,107,108]. Possible alterations in the properties and colloidal stability of NPs once released into an environment other than that of synthesis make studies under natural conditions difficult to interpret; thus, nanosafety assessments under reproducible and controlled conditions help to interpret investigations in ecotoxicological assays [24,86,109].

AuNPs, due to their unique intrinsic optical, biological, and catalytic properties [110,111,112] and their biocompatibility with mammalian systems, have been exploited in numerous medical and technological applications and used as model particles under laboratory conditions in nanotechnological research [113,114,115,116]. The effects on plants at the physiological and molecular level remain controversial [40,54,57,117]. In this light, this study aimed at studying the behavior of engineered AuNPs as a starting material and after dispersion in plant growth media, along with their physiological and molecular effects on the model plant *Arabidopsis thaliana*.

The high salt concentration of plant growth media may facilitate the aggregation of NPs and alterations in their bio-identity [118,119,120,121]; thus, surface-stabilizing agents are used to stabilize colloidal suspensions through electrostatic, steric, or electrosteric repulsion [122]. NP surface-stabilizing agents can play a key role in plant and animal toxicity tests [123,124]. In particular, the ionic charge conferred by particle coatings may influence the physical interaction between NPs and cell membranes, with positively charged NPs being more effective than negatively charged ones [125]. Barrena et al. [124] showed that, in germination tests of cucumber and lettuce seeds, toxic effects can be attributed to NP solvents rather than to NPs themselves. In this light, we showed that exposure of Arabidopsis seedlings to 10 mg/L of the negatively charged surface stabilizer SCTA did not influence Arabidopsis root growth.

Effects of electrostatically or sterically stabilized AuNPs have been studied in liquid and agar-solidified plant media, though their behavior and possible state of aggregation have not been further described in all cases [45,126,127,128]. By contrast, in their physiological and toxicological studies on Arabidopsis seedlings, Siegel et al. [41] dispersed SC-capped AuNPs in ^1^/_16_-diluted low-salinity MS, detecting a slight aggregation of AuNPs and consequently suboptimal conditions of plant growth assays. In this study, we tested the overtime stability of SC-stabilized and SCTA-stabilized AuNPs. The presence of traces of TA, the only difference between the two types of synthetized NPs, increased the stability of the particles by conferring a higher surface charge or partial steric stabilization and providing the necessary stability against salt-driven aggregation. A comprehensive physicochemical characterization of newly synthesized NPs, prior to and during their use in Arabidopsis treatments, was carried out, showing that the NPs are stable, dispersed, and usable for reproducible plant exposure experiments. 

Since contaminants in plant growth media allow for the growth of microorganisms, NPs need to be sterile before their use in plant assays. As shown by previous studies, autoclaving and radiation sterilization might result in the aggregation of the NPs, loss of the coating, and contamination with potential microbial toxins [129,130,131,132,133]. Sterile filtration has been shown not to directly affect the physical properties of NPs, but filter materials should be tested to exclude possible interactions with particle surfaces resulting in NP retention or coating removal [132]. We tested CME and PES filters and revealed that PES filters are suitable for sterilization of AuNPs, whereas CME filtering resulted in a significant reduction in the number of NPs in the filtered samples. Therefore, PES filters are considered suitable for AuNP sterilization to allow for sterile plant cultivation in the presence of AuNP-SCTA.

A number of studies have addressed AuNP responses in plants, reporting both positive and negative effects [40]. In this study, growth-promoting effects of AuNP-SCTA at a moderate concentration (10 mg/L) were revealed. As AuNP concentrations in the environment are very low, studies with lower concentrations might reflect more natural conditions [134]. Previous studies have found that AuNPs at high concentrations (≥100 mg/L) cause detrimental effects on plants, whereas for lower concentrations of AuNPs larger than 5 nm growth-promoting effects have been shown, supporting our findings that AuNPs have positive effects on plant growth [45,46,47,57]. Furthermore, Siegel et al. [41] tested three different sizes of AuNPs (10, 14, and 18 nm) at increasing concentrations (1, 10, and 100 mg/L) and showed that at the highest concentration the smaller particles reduced the length of the *Arabidopsis thaliana* root more than the larger ones. It has been hypothesized that a high concentration of NPs negatively affects plant growth by particle adsorption onto the cell wall of the root system, decreasing pore size and inhibiting water transport [124,135,136]. On the other hand, some contradictory studies have been reported. Feichtmeier et al. [135] reported a decrease in the biomass of *Hordeum vulgare* after AuNP treatment at a final concentration of between 3 and 10 mg/L. Some of these discrepancies can be explained by differences in specific experimental settings and different behavior of NPs under test conditions, which make a clear assessment of AuNP responses more difficult. Therefore, a careful evaluation of each study is necessary to draw a complete picture of the effects of AuNPs on plants.

Although the mechanisms of action and effects of AuNPs on plants are not yet fully understood [40,54,57,117], for the Au bulk counterpart the results are clearer. As plants have revealed their potential in the green synthesis of AuNPs, many studies have been produced on the physiological responses of plants to Au salts [137], used as starting material in order to obtain NPs. Gold is required by plants in traces, but its absorption in higher amounts can cause drastic changes in plant growth [40]. A previous study demonstrated that Arabidopsis seedlings treated with 10 mg/L of potassium tetrachloroaurate(III) (KaAuCl_4_) showed the formation of AuNPs in the roots and shoots and enhanced vegetative growth [138], whereas higher amounts of KAuCl_4_ or gold(III) chloride (AuCl_3_) (100 mg/L) negatively affected the root length and shoot development [139]. 

Immune responses are reported to be activated upon NP exposure in many plant and animal models, including reactive oxygen species (ROS) production and lipid peroxidation [48,140,141,142]. Here, we found that AuNP-SCTA alone did not induce these classical plant defense responses. In addition, ROS production induced by the 22-amino acid peptide derived from bacterial flagellin (flg22), sensed in plants as a pathogen-associated molecular pattern (PAMP), was significantly reduced in the presence of increasing amounts of AuNP-SCTA. To exclude a biophysical quenching effect of the light emitted by luminol, we performed lipid peroxidation assays. The same results were obtained by measuring lipid peroxidation, an indicator of oxidative stress in animals and plants [143], showing that the PAMP-triggered ROS burst was indeed reduced. Kumar et al. [47] showed that AuNPs at 80 mg/L significantly improved the free radical scavenging activity of Arabidopsis seedlings by increasing the activity of enzymes involved in the defense system against ROS, whereas plants treated with AuNPs in the range of 100 to 400 mg/L showed reduced growth, which was considered to be a consequence of increased free radical stress [40,144]. After 6 h of treatment with 10 mg/L of AuNP-SCTA, we found 10 peroxidases to be downregulated on the transcript level and 6 at the protein level, which were likely involved in oxidative stress reactions. These data indicate a correlation between ROS production and AuNP effects and show that AuNPs can reduce stress responses triggered by immune stimulatory peptides. Whether this effect is based on a direct effect on the peptide, e.g., through adsorption to the NP surface or changes in the peptide accessibility (and consequent alteration of its mode of perception) [145], or on a protective effect of AuNPs on PAMP recognition or downstream signaling, will be interesting to study in the future.

We performed transcriptomics and proteomics analyses to study the alterations caused by short (6 h) and long (7 d) AuNP exposure at the molecular level. In particular, after short-term AuNP treatment, genes involved in disease resistance, defense response, oxidative stress, and auxin- and metal-response were downregulated. In the proteomic analysis after short treatment we detected two distinct categories of upregulated proteins, i.e., proteins involved in responses to oxidative stress and abiotic stimuli, whereas after long NP exposure all upregulated proteins were annotated as involved in development processes. To our knowledge, this is the only study analyzing the transcriptomic and proteomic changes after AuNP treatments in Arabidopsis, whereas such analyses have been performed on the roots of Arabidopsis seedlings upon gold (KAuCl_4_) exposure, which leads to AuNP formation by the plant [138]. As for the physiological effects mentioned above, at the molecular level the changes induced by gold exposure also showed some effects similar to those induced by AuNP exposure. A comparative analysis between our transcriptomics data and Tiwari et al.’s [138] shows that there is a significant overlap of up- and downregulated genes (three common upregulated and 22 common downregulated genes, Figure S7). In particular, between the upregulated DEGs two metal response genes (MT1C, At1g07610 and ALMT1, At1g08430) and DGR2 (At5g25460) were found. In both studies, disease and defense response and oxidative stress genes were downregulated. By contrast, Tiwari et al. [138] found that developmental, auxin-responsive, and metal-responsive genes were upregulated after Au treatment, whereas in our study the same categories of genes were downregulated after AuNP treatment. These differences were likely caused by different effects caused by Au ion uptake compared to exposure to nanoparticles. As metal AuNPs are very inert, the significant overlap between Au salt and AuNP is potentially caused by NP effects in both experiments, as Au ions are taken up and converted into AuNPs inside the plant, where they may cause similar effects as external NP exposure.

An overlap analysis of our proteomic and transcriptomic studies revealed two particularly interesting candidates: DUF642 L-GalL-responsive genes 1 and 2 (DGR1, At1g80240 and DGR2, At5g25460), which were also found to be upregulated by Au salt exposure [138]. DGR1 and DGR2 encode for two proteins belonging to the DUF642 protein family, whose members are part of the cell wall proteome [96] and have shown in Arabidopsis a complementary expression pattern in young and developed roots, suggesting a similar but non-redundant function [95]. As Gao et al. reported in their study [95], DGRs are involved in the development processes of Arabidopsis, and in particular in root elongation. DGR2 seems to have a predominant role, as *dgr2* single mutants show a short, undeveloped root phenotype [95]. These results suggest the potential involvement of these proteins in the root growth-promoting effects induced by AuNPs and can be used as a starting point for further studies aimed at dissecting the pathways underlying the beneficial effects of AuNP-SCTA on Arabidopsis development.

## 5. Conclusions

**Figure 7:**
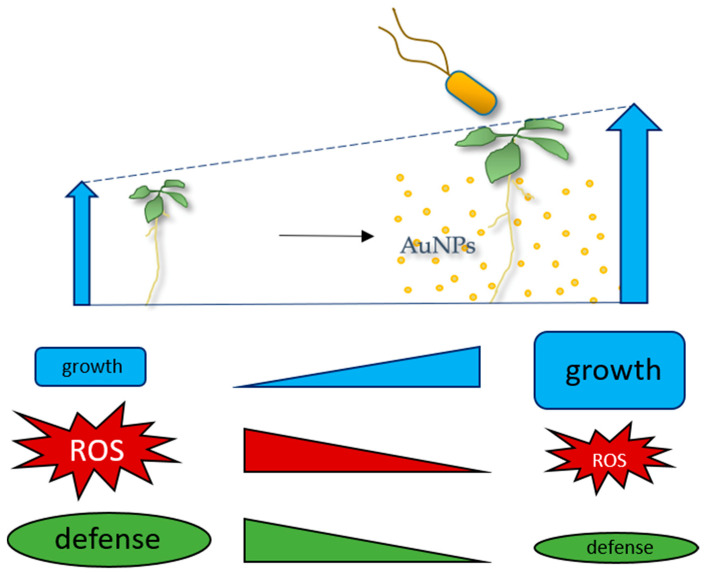
Model of AuNP effects on Arabidopsis seedlings. AuNPs stabilized with SCTA have growth-promoting effects on Arabidopsis seedlings and can reduce oxidative stress genes/proteins and ROS burst after triggering with the pathogen-associated molecular pattern (PAMP) flg22, indicating that the NPs can shift the trade-off between growth and defense responses to the growth side.

NPs are released into the environment in increasing amounts and their high reactivity may cause problems that are not associated with the respective bulk material. Therefore, an ecotoxicological assessment is necessary to evaluate their risk in nature, but to understand the molecular mechanisms underlying the effects of NPs on the environment, controlled model systems are necessary. Here, we describe the establishment of a stable and reproducible system to study plant responses to AuNPs after short- and long-term exposure. Both initial and overtime characterization of NPs, especially after dispersal in new environments, is essential. The effects resulting from NP-plant interaction need stable, sterile, and reproducible colloidal solutions, ensured by the use of non-toxic NP surface stabilizing agents. In this study, we demonstrated that these AuNP-SCTAs positively influence the growth of Arabidopsis seedlings, while also conferring partial protection against oxidative stress caused by triggering immune-responses. Transcriptomics and proteomics studies show downregulation of (oxidative) stress and immune responses and upregulation of growth-promoting genes and support the scenario that the trade-off between growth and immune/stress responses are shifted to the growth side after AuNP exposure (Figure 7). The identified DEGs and DEPs provide a useful data source for future analysis of the molecular mechanism underlying AuNP-induced growth stimulation.

## Supplementary Materials

The following information is available online at https://www.mdpi.com/article/10.3390/nano11123161/s1, Figure S1: Physicochemical characterization of AuNP-SC dispersed in H2O, ½ MS, and ½ MS agar; Figure S2: Growth of Arabidopsis seedlings in the absence and presence of AuNPs; Figure S3: GO classification of DEGs after AuNP-SCTA treatment; Figure S4: Pathway classification of DEGs after AuNP-SCTA treatment; Figure S5: GO classification of DEPs after AuNP-SCTA treatment; Figure S6: Pathway classification of DEPs after AuNP-SCTA treatment; Figure S7: Venn diagram of DEGs after AuNP-SCTA exposure (this study) and DEG after exposure to KAuCl4 resulting in in planta AuNP formation [140]. Table of the overlap between our DEGs and those published by Tiwari et al. (2016); Table S1: Clean reads quality metrics and summary of genome mapping; Table S2: DEGs list after short (6 h) and long (7 d) AuNP-SCTA treatment; Table S3: DEP list after short (6 h) and long (7 d) AuNP-SCTA treatment; Table S4: Transcriptomic and proteomic studies: overview and overlap.

## References

[1] Bundschuh M., Filser J., Lüderwald S., McKee M.S., Metreveli G., Schaumann G.E., Schulz R., Wagner S. (2018). Nanoparticles in the environment: Where do we come from, where do we go to?. Environ. Sci. Eur..

[2] Nowack B., Bucheli T.D. (2007). Occurrence, behavior and effects of nanoparticles in the environment. Environ. Pollut..

[3] Lespes G., Faucher S., Slaveykova V.I. (2020). Natural Nanoparticles, Anthropogenic Nanoparticles, Where Is the Frontier?. Front. Environ. Sci..

[4] Hochella M.F., Mogk D.W., Ranville J., Allen I.C., Luther G.W., Marr L.C., McGrail B.P., Murayama M., Qafoku N.P., Rosso K.M. (2019). Natural, incidental, and engineered nanomaterials and their impacts on the Earth system. Science.

[5] Boraschi D., Alijagic A., Auguste M., Barbero F., Ferrari E., Hernadi S., Mayall C., Michelini S., Navarro Pacheco N.I., Prinelli A. (2020). Addressing Nanomaterial Immunosafety by Evaluating Innate Immunity across Living Species. Small.

[6] Ramalingam V. (2019). Multifunctionality of gold nanoparticles: Plausible and convincing properties. Adv. Colloid Interface Sci..

[7] Alaqad K., Saleh T. (2016). Gold and Silver Nanoparticles: Synthesis Methods, Characterization Routes and Applications towards Drugs. J. Environ. Anal. Toxicol..

[8] Leso V., Fontana L., Iavicoli I. (2019). Biomedical nanotechnology: Occupational views. Nano Today.

[9] Ashraf R., Amna T., Sheikh F.A., Sheikh F.A. (2020). Unique Properties of the Gold Nanoparticles: Synthesis, Functionalization and Applications. Application of Nanotechnology in Biomedical Sciences.

[10] Spampinato V., Parracino M.A., La Spina R., Rossi F., Ceccone G. (2016). Surface Analysis of Gold Nanoparticles Functionalized with Thiol-Modified Glucose SAMs for Biosensor Applications. Front. Chem..

[11] Singh P., Pandit S., Mokkapati V.R.S.S., Garg A., Ravikumar V., Mijakovic I. (2018). Gold Nanoparticles in Diagnostics and Therapeutics for Human Cancer. Int. J. Mol. Sci..

[12] Grisel R., Weststrate K.-J., Gluhoi A., Nieuwenhuys B.E. (2002). Catalysis by Gold Nanoparticles. Gold Bull..

[13] Ojea-Jiménez I., López X., Arbiol J., Puntes V. (2012). Citrate-Coated Gold Nanoparticles As Smart Scavengers for Mercury(II) Removal from Polluted Waters. ACS Nano.

[14] Hua Z., Yu T., Liu D., Xianyu Y. (2021). Recent advances in gold nanoparticles-based biosensors for food safety detection. Biosens. Bioelectron..

[15] Li L., Zhang M., Chen W. (2020). Gold nanoparticle-based colorimetric and electrochemical sensors for the detection of illegal food additives. J. Food Drug Anal..

[16] Mittal D., Kaur G., Singh P., Yadav K., Ali S.A. (2020). Nanoparticle-Based Sustainable Agriculture and Food Science: Recent Advances and Future Outlook. Front. Nanotechnol..

[17] Sundararajan B., Ranjitha Kumari B.D. (2017). Novel synthesis of gold nanoparticles using *Artemisia vulgaris* L. leaf extract and their efficacy of larvicidal activity against dengue fever vector *Aedes aegypti* L.. J. Trace Elem. Med. Biol..

[18] Thakur R.K., Dhirta B., Shirkot P. (2018). Studies on effect of gold nanoparticles on Meloidogyne incognita and tomato plants growth and development. bioRxiv.

[19] Giese B., Klaessig F., Park B., Kaegi R., Steinfeldt M., Wigger H., von Gleich A., Gottschalk F. (2018). Risks, Release and Concentrations of Engineered Nanomaterial in the Environment. Sci. Rep..

[20] Yokel R.A., Macphail R.C. (2011). Engineered nanomaterials: Exposures, hazards, and risk prevention. J. Occup. Med. Toxicol..

[21] Mourdikoudis S., Pallares R.M., Thanh N.T.K. (2018). Characterization techniques for nanoparticles: Comparison and complementarity upon studying nanoparticle properties. Nanoscale.

[22] Albanese A., Tang P.S., Chan W.C. (2012). The effect of nanoparticle size, shape, and surface chemistry on biological systems. Annu. Rev. Biomed. Eng..

[23] Powers K., Palazuelos M., Moudgil B., Roberts S. (2009). Characterization of the size, shape, and state of dispersion of nanoparticles for toxicological studies. Nanotoxicology.

[24] Barbero F., Moriones O.H., Bastús N.G., Puntes V. (2019). Dynamic Equilibrium in the Cetyltrimethylammonium Bromide–Au Nanoparticle Bilayer, and the Consequent Impact on the Formation of the Nanoparticle Protein Corona. Bioconjug. Chem..

[25] Ai H., Jones S.A., Lvov Y.M. (2003). Biomedical applications of electrostatic layer-by-layer nano-assembly of polymers, enzymes, and nanoparticles. Cell Biochem. Biophys..

[26] Wagner S., Gondikas A., Neubauer E., Hofmann T., von der Kammer F. (2014). Spot the Difference: Engineered and Natural Nanoparticles in the Environment—Release, Behavior, and Fate. Angew. Chem. Int. Ed..

[27] Wu H., Huang L., Rose A., Grassian V.H. (2020). Impact of surface adsorbed biologically and environmentally relevant coatings on TiO_2_ nanoparticle reactivity. Environ. Sci. Nano.

[28] Lopez-Chaves C., Soto-Alvaredo J., Montes-Bayon M., Bettmer J., Llopis J., Sanchez-Gonzalez C. (2018). Gold nanoparticles: Distribution, bioaccumulation and toxicity. In vitro and in vivo studies. Nanomedicine.

[29] Downs T.R., Crosby M.E., Hu T., Kumar S., Sullivan A., Sarlo K., Reeder B., Lynch M., Wagner M., Mills T. (2012). Silica nanoparticles administered at the maximum tolerated dose induce genotoxic effects through an inflammatory reaction while gold nanoparticles do not. Mutat. Res. Genet. Toxicol. Environ. Mutagen..

[30] Khan H.A., Abdelhalim M.A.K., Alhomida A.S., Al-Ayed M.S. (2013). Effects of Naked Gold Nanoparticles on Proinflammatory Cytokines mRNA Expression in Rat Liver and Kidney. BioMed Res. Int..

[31] Yang C., Yang H., Wu J., Meng Z., Xing R., Tian A., Tian X., Guo L., Zhang Y., Nie G. (2013). No overt structural or functional changes associated with PEG-coated gold nanoparticles accumulation with acute exposure in the mouse heart. Toxicol. Lett..

[32] Piryazev A.P., Azizova O.A., Aseichev A.V., Dudnik L.B., Sergienko V.I. (2013). Effect of gold nanoparticles on production of reactive oxygen species by human peripheral blood leukocytes stimulated with opsonized zymosan. Bull. Exp. Biol. Med..

[33] Mateo D., Morales P., Ávalos A., Haza A.I. (2014). Oxidative stress contributes to gold nanoparticle-induced cytotoxicity in human tumor cells. Toxicol. Mech. Methods.

[34] Li J.J., Hartono D., Ong C.-N., Bay B.-H., Yung L.-Y.L. (2010). Autophagy and oxidative stress associated with gold nanoparticles. Biomaterials.

[35] Li T., Albee B., Alemayehu M., Diaz R., Ingham L., Kamal S., Rodriguez M., Bishnoi S.W. (2010). Comparative toxicity study of Ag, Au, and Ag-Au bimetallic nanoparticles on Daphnia magna. Anal. Bioanal. Chem..

[36] Falagan-Lotsch P., Grzincic E.M., Murphy C.J. (2016). One low-dose exposure of gold nanoparticles induces long-term changes in human cells. Proc. Natl. Acad. Sci. USA.

[37] Sousa A.A., Hassan S.A., Knittel L.L., Balbo A., Aronova M.A., Brown P.H., Schuck P., Leapman R.D. (2016). Biointeractions of ultrasmall glutathione-coated gold nanoparticles: Effect of small size variations. Nanoscale.

[38] Tiede K., Hassellöv M., Breitbarth E., Chaudhry Q., Boxall A.B.A. (2009). Considerations for environmental fate and ecotoxicity testing to support environmental risk assessments for engineered nanoparticles. J. Chromatogr. A.

[39] Batley G.E., Kirby J.K., McLaughlin M.J. (2013). Fate and Risks of Nanomaterials in Aquatic and Terrestrial Environments. Acc. Chem. Res..

[40] Siddiqi K., Husen A. (2016). Engineered Gold Nanoparticles and Plant Adaptation Potential. Nanoscale Res. Lett..

[41] Siegel J., Záruba K., Švorčík V., Kroumanová K., Burketová L., Martinec J. (2018). Round-shape gold nanoparticles: Effect of particle size and concentration on *Arabidopsis thaliana* root growth. Nanoscale Res. Lett..

[42] Pan Y., Leifert A., Ruau D., Neuss S., Bornemann J., Schmid G., Brandau W., Simon U., Jahnen-Dechent W. (2009). Gold Nanoparticles of Diameter 1.4 nm Trigger Necrosis by Oxidative Stress and Mitochondrial Damage. Small.

[43] Coradeghini R., Gioria S., García C.P., Nativo P., Franchini F., Gilliland D., Ponti J., Rossi F. (2013). Size-dependent toxicity and cell interaction mechanisms of gold nanoparticles on mouse fibroblasts. Toxicol. Lett..

[44] Boyoglu C., He Q., Willing G., Boyoglu-Barnum S., Dennis V.A., Pillai S., Singh S.R. (2013). Microscopic Studies of Various Sizes of Gold Nanoparticles and Their Cellular Localizations. ISRN Nanomater..

[45] Arora S., Sharma P., Kumar S., Nayan R., Khanna P.K., Zaidi M.G.H. (2012). Gold-nanoparticle induced enhancement in growth and seed yield of Brassica juncea. Plant Growth Regul..

[46] Mahakham W., Theerakulpisut P., Maensiri S., Phumying S., Sarmah A. (2016). Environmentally benign synthesis of phytochemicals-capped gold nanoparticles as nanopriming agent for promoting maize seed germination. Sci. Total Environ..

[47] Kumar V., Guleria P., Kumar V., Yadav S.K. (2013). Gold nanoparticle exposure induces growth and yield enhancement in *Arabidopsis thaliana*. Sci. Total Environ..

[48] Marslin G., Sheeba C., Gregory F. (2017). Nanoparticles Alter Secondary Metabolism in Plants via ROS Burst. Front. Plant Sci..

[49] Chandra S., Chakraborty N., Dasgupta A., Sarkar J., Panda K., Acharya K. (2015). Chitosan nanoparticles: A positive modulator of innate immune responses in plants. Sci. Rep..

[50] Iqbal M.S., Singh A.K., Singh S.P., Ansari M.I., Bhushan I., Singh V.K., Tripathi D.K. (2020). Nanoparticles and Plant Interaction with Respect to Stress Response. Nanomaterials and Environmental Biotechnology.

[51] Yang L., Kuang H., Zhang W., Aguilar Z.P., Wei H., Xu H. (2017). Comparisons of the biodistribution and toxicological examinations after repeated intravenous administration of silver and gold nanoparticles in mice. Sci. Rep..

[52] Zhao L., Lu L., Aodi W., Zhang H., Huang M., Wu H., Xing B., Wang Z., Ji R. (2020). Nanobiotechnology in Agriculture: Use of Nanomaterials To Promote Plant Growth and Stress Tolerance. J. Agric. Food Chem..

[53] Milewska-Hendel A., Zubko M., Karcz J., Stróż D., Kurczyńska E. (2017). Fate of neutral-charged gold nanoparticles in the roots of the *Hordeum vulgare* L. cultivar Karat. Sci. Rep..

[54] Zhu Z.J., Wang H., Yan B., Zheng H., Jiang Y., Miranda O.R., Rotello V.M., Xing B., Vachet R.W. (2012). Effect of surface charge on the uptake and distribution of gold nanoparticles in four plant species. Environ. Sci. Technol..

[55] Koelmel J., Leland T., Wang H., Amarasiriwardena D., Xing B. (2013). Investigation of gold nanoparticles uptake and their tissue level distribution in rice plants by laser ablation-inductively coupled-mass spectrometry. Environ. Pollut..

[56] Judy J.D., Unrine J.M., Rao W., Wirick S., Bertsch P.M. (2012). Bioavailability of Gold Nanomaterials to Plants: Importance of Particle Size and Surface Coating. Environ. Sci. Technol..

[57] Sabo-Attwood T., Unrine J.M., Stone J.W., Murphy C.J., Ghoshroy S., Blom D., Bertsch P.M., Newman L.A. (2012). Uptake, distribution and toxicity of gold nanoparticles in tobacco (Nicotiana xanthi) seedlings. Nanotoxicology.

[58] Carpita N., Sabularse D., Montezinos D., Delmer D.P. (1979). Determination of the Pore Size of Cell Walls of Living Plant Cells. Science.

[59] García-Sánchez S., Bernales I., Cristobal S. (2015). Early response to nanoparticles in the Arabidopsis transcriptome compromises plant defence and root-hair development through salicylic acid signalling. BMC Genom..

[60] Kaveh R., Li Y.S., Ranjbar S., Tehrani R., Brueck C.L., Van Aken B. (2013). Changes in *Arabidopsis thaliana* gene expression in response to silver nanoparticles and silver ions. Environ. Sci. Technol..

[61] Zhang C.L., Jiang H.S., Gu S.P., Zhou X.H., Lu Z.W., Kang X.H., Yin L., Huang J. (2019). Combination analysis of the physiology and transcriptome provides insights into the mechanism of silver nanoparticles phytotoxicity. Environ. Pollut..

[62] Tumburu L., Andersen C.P., Rygiewicz P.T., Reichman J.R. (2017). Molecular and physiological responses to titanium dioxide and cerium oxide nanoparticles in Arabidopsis. Environ. Toxicol. Chem..

[63] Simon D.F., Domingos R.F., Hauser C., Hutchins C.M., Zerges W., Wilkinson K.J. (2013). Transcriptome sequencing (RNA-seq) analysis of the effects of metal nanoparticle exposure on the transcriptome of *Chlamydomonas reinhardtii*. Appl. Environ. Microbiol..

[64] Bastús N.G., Comenge J., Puntes V. (2011). Kinetically controlled seeded growth synthesis of citrate-stabilized gold nanoparticles of up to 200 nm: Size focusing versus Ostwald ripening. Langmuir.

[65] Piella J., Bastús N.G., Puntes V. (2016). Size-Controlled Synthesis of Sub-10-nanometer Citrate-Stabilized Gold Nanoparticles and Related Optical Properties. Chem. Mater..

[66] Chen Y., Xianyu Y., Jiang X. (2017). Surface Modification of Gold Nanoparticles with Small Molecules for Biochemical Analysis. Acc. Chem. Res..

[67] Ghosh P., Han G., De M., Kim C.K., Rotello V.M. (2008). Gold nanoparticles in delivery applications. Adv. Drug Deliv. Rev..

[68] Hvolbæk B., Janssens T.V.W., Clausen B.S., Falsig H., Christensen C.H., Nørskov J.K. (2007). Catalytic activity of Au nanoparticles. Nano Today.

[69] Albert M., Butenko M.A., Aalen R.B., Felix G., Wildhagen M. (2015). Chemiluminescence Detection of the Oxidative Burst in Plant Leaf Pieces. Bio-protocol.

[70] Hermes-Lima M., Willmore W.G., Storey K.B. (1995). Quantification of lipid peroxidation in tissue extracts based on Fe(III)xylenol orange complex formation. Free Radic. Biol. Med..

[71] Schmieg H., Huppertsberg S., Knepper T.P., Krais S., Reitter K., Rezbach F., Ruhl A.S., Köhler H.-R., Triebskorn R. (2020). Polystyrene microplastics do not affect juvenile brown trout (Salmo trutta f. fario) or modulate effects of the pesticide methiocarb. Environ. Sci. Eur..

[72] Kim D., Langmead B., Salzberg S.L. (2015). HISAT: A fast spliced aligner with low memory requirements. Nat. Methods.

[73] Pertea M., Pertea G.M., Antonescu C.M., Chang T.-C., Mendell J.T., Salzberg S.L. (2015). StringTie enables improved reconstruction of a transcriptome from RNA-seq reads. Nat. Biotechnol..

[74] Trapnell C., Roberts A., Goff L., Pertea G., Kim D., Kelley D.R., Pimentel H., Salzberg S.L., Rinn J.L., Pachter L. (2012). Differential gene and transcript expression analysis of RNA-seq experiments with TopHat and Cufflinks. Nat. Protoc..

[75] Kong L., Zhang Y., Ye Z.Q., Liu X.Q., Zhao S.Q., Wei L., Gao G. (2007). CPC: Assess the protein-coding potential of transcripts using sequence features and support vector machine. Nucleic Acids Res..

[76] Langmead B., Salzberg S.L. (2012). Fast gapped-read alignment with Bowtie 2. Nat. Methods.

[77] Li B., Dewey C.N. (2011). RSEM: Accurate transcript quantification from RNA-Seq data with or without a reference genome. BMC Bioinform..

[78] Tarazona S., García-Alcalde F., Dopazo J., Ferrer A., Conesa A. (2011). Differential expression in RNA-seq: A matter of depth. Genome Res.

[79] Borchert N., Dieterich C., Krug K., Schütz W., Jung S., Nordheim A., Sommer R.J., Macek B. (2010). Proteogenomics of Pristionchus pacificus reveals distinct proteome structure of nematode models. Genome Res..

[80] Rappsilber J., Mann M., Ishihama Y. (2007). Protocol for micro-purification, enrichment, pre-fractionation and storage of peptides for proteomics using StageTips. Nat. Protoc..

[81] Boersema P.J., Raijmakers R., Lemeer S., Mohammed S., Heck A.J.R. (2009). Multiplex peptide stable isotope dimethyl labeling for quantitative proteomics. Nat. Protoc..

[82] Kliza K., Taumer C., Pinzuti I., Franz-Wachtel M., Kunzelmann S., Stieglitz B., Macek B., Husnjak K. (2017). Internally tagged ubiquitin: A tool to identify linear polyubiquitin-modified proteins by mass spectrometry. Nat. Methods.

[83] Cox J., Mann M. (2008). MaxQuant enables high peptide identification rates, individualized p.p.b.-range mass accuracies and proteome-wide protein quantification. Nat. Biotechnol..

[84] Elias J.E., Gygi S.P. (2007). Target-decoy search strategy for increased confidence in large-scale protein identifications by mass spectrometry. Nat. Methods.

[85] Tyanova S., Temu T., Sinitcyn P., Carlson A., Hein M.Y., Geiger T., Mann M., Cox J. (2016). The Perseus computational platform for comprehensive analysis of (prote)omics data. Nat. Methods.

[86] Barbero F., Russo L., Vitali M., Piella J., Salvo I., Borrajo M.L., Busquets-Fité M., Grandori R., Bastús N.G., Casals E. (2017). Formation of the Protein Corona: The Interface between Nanoparticles and the Immune System. Semin. Immunol..

[87] Sepúlveda B., Angelomé P.C., Lechuga L.M., Liz-Marzán L.M. (2009). LSPR-based nanobiosensors. Nano Today.

[88] Cosgrove T. (2010). Colloid Science: Principles, Methods and Applications.

[89] Abbott S., Holmes N. (2013). Nanocoatings: Principles and Practice: From Research to Production.

[90] Wilson-Corral V., Anderson C., Rodriguez M. (2012). Gold phytomining. A review of the relevance of this technology to mineral extraction in the 21st century. J. Environ. Manag..

[91] Saijo Y., Loo E., Yasuda S. (2017). Pattern recognition receptors and signaling in plant-microbe interactions. Plant J..

[92] Farmer E.E., Mueller M.J. (2013). ROS-mediated lipid peroxidation and RES-activated signaling. Annu. Rev. Plant Biol..

[93] Ranf S. (2016). Immune Sensing of Lipopolysaccharide in Plants and Animals: Same but Different. PLoS Pathog..

[94] Su L.-J., Zhang J.-H., Gomez H., Murugan R., Hong X., Xu D., Jiang F., Peng Z.-Y. (2019). Reactive Oxygen Species-Induced Lipid Peroxidation in Apoptosis, Autophagy, and Ferroptosis. Oxid. Med. Cell. Longev..

[95] Gao Y., Badejo A.A., Sawa Y., Ishikawa T. (2012). Analysis of two L-Galactono-1,4-lactone-responsive genes with complementary expression during the development of *Arabidopsis thaliana*. Plant Cell Physiol..

[96] Cruz-Valderrama J.E., Gómez-Maqueo X., Salazar-Iribe A., Zúñiga-Sánchez E., Hernández-Barrera A., Quezada-Rodríguez E., Gamboa-deBuen A. (2019). Overview of the Role of Cell Wall DUF642 Proteins in Plant Development. Int. J. Mol. Sci..

[97] Klatte M., Schuler M., Wirtz M., Fink-Straube C., Hell R., Bauer P. (2009). The Analysis of Arabidopsis Nicotianamine Synthase Mutants Reveals Functions for Nicotianamine in Seed Iron Loading and Iron Deficiency Responses. Plant Physiol..

[98] Gupta R., Xie H. (2018). Nanoparticles in Daily Life: Applications, Toxicity and Regulations. J. Environ. Pathol. Toxicol. Oncol..

[99] Liu J., Williams P.C., Geisler-Lee J., Goodson B.M., Fakharifar M., Peiravi M., Chen D., Lightfoot D.A., Gemeinhardt M.E. (2018). Impact of wastewater effluent containing aged nanoparticles and other components on biological activities of the soil microbiome, Arabidopsis plants, and earthworms. Environ. Res..

[100] Singh D., Kumar A. (2014). Human Exposures of Engineered Nanoparticles from Plants Irrigated with Contaminated Water: Mixture Toxicity Issues and Challenges Ahead. Adv. Sci. Lett..

[101] Stampoulis D., Sinha S.K., White J.C. (2009). Assay-Dependent Phytotoxicity of Nanoparticles to Plants. Environ. Sci. Technol..

[102] Chawla J., Singh D., Sundaram B., Kumar A. (2018). Identifying Challenges in Assessing Risks of Exposures of Silver Nanoparticles. Expo. Health.

[103] Zia-ur-Rehman M., Qayyum M.F., Akmal F., Maqsood M.A., Rizwan M., Waqar M., Azhar M., Tripathi D.K., Ahmad P., Sharma S., Chauhan D.K., Dubey N.K. (2018). Chapter 7—Recent Progress of Nanotoxicology in Plants. Nanomaterials in Plants, Algae, and Microorganisms.

[104] Sanzari I., Leone A., Ambrosone A. (2019). Nanotechnology in Plant Science: To Make a Long Story Short. Front. Bioeng. Biotechnol..

[105] Kranjc E., Drobne D. (2019). Nanomaterials in Plants: A Review of Hazard and Applications in the Agri-Food Sector. Nanomaterials.

[106] Du W., Xu Y., Yin Y., Ji R., Guo H. (2018). Risk assessment of engineered nanoparticles and other contaminants in terrestrial plants. Curr. Opin. Environ. Sci. Health.

[107] Khan I., Saeed K., Khan I. (2019). Nanoparticles: Properties, applications and toxicities. Arab. J. Chem..

[108] Sukhanova A., Bozrova S., Sokolov P., Berestovoy M., Karaulov A., Nabiev I. (2018). Dependence of Nanoparticle Toxicity on Their Physical and Chemical Properties. Nanoscale Res. Lett..

[109] Kim T., Lee C.H., Joo S.W., Lee K. (2008). Kinetics of gold nanoparticle aggregation: Experiments and modeling. J. Colloid Interface Sci.

[110] Hu X., Zhang Y., Ding T., Liu J., Zhao H. (2020). Multifunctional Gold Nanoparticles: A Novel Nanomaterial for Various Medical Applications and Biological Activities. Front. Bioeng. Biotechnol..

[111] Das M., Shim K.H., An S.S.A., Yi D.K. (2011). Review on gold nanoparticles and their applications. Toxicol. Environ. Health Sci..

[112] Zhang X. (2015). Gold Nanoparticles: Recent Advances in the Biomedical Applications. Cell Biochem. Biophys..

[113] Azzazy H., Mansour M., Samir T., Franco R. (2011). Gold nanoparticles in the clinical laboratory: Principles of preparation and applications. Clin. Chem. Lab. Med..

[114] Connor E.E., Mwamuka J., Gole A., Murphy C.J., Wyatt M.D. (2005). Gold Nanoparticles Are Taken Up by Human Cells but Do Not Cause Acute Cytotoxicity. Small.

[115] Sperling R.A., Rivera Gil P., Zhang F., Zanella M., Parak W.J. (2008). Biological applications of gold nanoparticles. Chem. Soc. Rev..

[116] Li Y., Italiani P., Casals E., Valkenborg D., Mertens I., Baggerman G., Nelissen I., Puntes V.F., Boraschi D. (2016). Assessing the Immunosafety of Engineered Nanoparticles with a Novel in Vitro Model Based on Human Primary Monocytes. ACS Appl. Mater. Interfaces.

[117] Rico C.M., Majumdar S., Duarte-Gardea M., Peralta-Videa J.R., Gardea-Torresdey J.L. (2011). Interaction of Nanoparticles with Edible Plants and Their Possible Implications in the Food Chain. J. Agric. Food Chem..

[118] Fuller M., Köper I. (2018). Polyelectrolyte-Coated Gold Nanoparticles: The Effect of Salt and Polyelectrolyte Concentration on Colloidal Stability. Polymers.

[119] Sun M., Liu F., Zhu Y., Wang W., Hu J., Liu J., Dai Z., Wang K., Wei Y., Bai J. (2016). Salt-Induced Aggregation of Gold Nanoparticles for Photoacoustic Imaging and Photothermal Therapy of Cancer. Nanoscale.

[120] Nierenberg D., Khaled A.R., Flores O. (2018). Formation of a protein corona influences the biological identity of nanomaterials. Rep. Pract. Oncol. Radiother..

[121] Moore T., Rodriguez Lorenzo L., Hirsch V., Balog S., Urban D., Jud C., Rothen-Rutishauser B., Lattuada M., Fink A. (2015). Nanoparticle colloidal stability in cell culture media and impact on cellular interactions. Chem. Soc. Rev..

[122] Guerrini L., Alvarez-Puebla R.A., Pazos-Perez N. (2018). Surface Modifications of Nanoparticles for Stability in Biological Fluids. Materials.

[123] Attarilar S., Yang J., Ebrahimi M., Wang Q., Liu J., Tang Y., Yang J. (2020). The Toxicity Phenomenon and the Related Occurrence in Metal and Metal Oxide Nanoparticles: A Brief Review From the Biomedical Perspective. Front. Bioeng. Biotechnol..

[124] Barrena R., Casals E., Colón J., Font X., Sánchez A., Puntes V. (2009). Evaluation of the ecotoxicity of model nanoparticles. Chemosphere.

[125] El Badawy A.M., Silva R.G., Morris B., Scheckel K.G., Suidan M.T., Tolaymat T.M. (2011). Surface Charge-Dependent Toxicity of Silver Nanoparticles. Environ. Sci. Technol..

[126] Avellan A., Schwab F., Masion A., Chaurand P., Borschneck D., Vidal V., Rose J., Santaella C., Levard C. (2017). Nanoparticle Uptake in Plants: Gold Nanomaterial Localized in Roots of *Arabidopsis thaliana* by X-ray Computed Nanotomography and Hyperspectral Imaging. Environ. Sci. Technol..

[127] Lovecká P., Macůrková A., Záruba K., Hubáček T., Siegel J., Valentová O. (2021). Genomic Damage Induced in *Nicotiana tabacum* L. Plants by Colloidal Solution with Silver and Gold Nanoparticles. Plants.

[128] Milewska-Hendel A., Zubko M., Stróż D., Kurczyńska E.U. (2019). Effect of Nanoparticles Surface Charge on the *Arabidopsis thaliana* (L.) Roots Development and Their Movement into the Root Cells and Protoplasts. Int. J. Mol. Sci..

[129] Masson V., Maurin F., Fessi H., Devissaguet J.P. (1997). Influence of sterilization processes on poly(epsilon-caprolactone) nanospheres. Biomaterials.

[130] Özcan I., Bouchemal K., Segura Sánchez F., Abaci Ö., Özer Ö., Güneri T., Ponchel G. (2009). Effects of sterilization techniques on the PEGylated poly (γ-benzyl-L-glutamate) (PBLG) nanoparticles. Acta Pharm. Sci..

[131] Memisoglu-Bilensoy E., Hincal A.A. (2006). Sterile, injectable cyclodextrin nanoparticles: Effects of gamma irradiation and autoclaving. Int. J. Pharm..

[132] Bernal-Chávez S.A., Del Prado-Audelo M.L., Caballero-Florán I.H., Giraldo-Gomez D.M., Figueroa-Gonzalez G., Reyes-Hernandez O.D., González-Del Carmen M., González-Torres M., Cortés H., Leyva-Gómez G. (2021). Insights into Terminal Sterilization Processes of Nanoparticles for Biomedical Applications. Molecules.

[133] França Á., Pelaz B., Moros M., Sánchez-Espinel C., Hernández A., Fernández-López C., Grazú V., de la Fuente J.M., Pastoriza-Santos I., Liz-Marzán L.M. (2010). Sterilization Matters: Consequences of Different Sterilization Techniques on Gold Nanoparticles. Small.

[134] Mahapatra I., Sun T.Y., Clark J.R.A., Dobson P.J., Hungerbuehler K., Owen R., Nowack B., Lead J. (2015). Probabilistic modelling of prospective environmental concentrations of gold nanoparticles from medical applications as a basis for risk assessment. J. Nanobiotechnol..

[135] Feichtmeier N.S., Walther P., Leopold K. (2015). Uptake, effects, and regeneration of barley plants exposed to gold nanoparticles. Environ. Sci. Pollut. Res. Int..

[136] Asli S., Neumann P.M. (2009). Colloidal suspensions of clay or titanium dioxide nanoparticles can inhibit leaf growth and transpiration via physical effects on root water transport. Plant Cell Environ..

[137] Khan T., Ullah N., Khan M.A., Mashwani Z.-u.-R., Nadhman A. (2019). Plant-based gold nanoparticles; a comprehensive review of the decade-long research on synthesis, mechanistic aspects and diverse applications. Adv. Colloid Interface Sci..

[138] Tiwari M., Krishnamurthy S., Shukla D., Kiiskila J., Jain A., Datta R., Sharma N., Sahi S.V. (2016). Comparative transcriptome and proteome analysis to reveal the biosynthesis of gold nanoparticles in Arabidopsis. Sci. Rep..

[139] Taylor A.F., Rylott E.L., Anderson C.W.N., Bruce N.C. (2014). Investigating the Toxicity, Uptake, Nanoparticle Formation and Genetic Response of Plants to Gold. PLoS ONE.

[140] Abdal Dayem A., Hossain M.K., Lee S.B., Kim K., Saha S.K., Yang G.-M., Choi H.Y., Cho S.-G. (2017). The Role of Reactive Oxygen Species (ROS) in the Biological Activities of Metallic Nanoparticles. Int. J. Mol. Sci..

[141] Feidantsis K., Kalogiannis S., Marinoni A., Vasilogianni A.M., Gkanatsiou C., Kastrinaki G., Dendrinou-Samara C., Kaloyianni M. (2020). Toxicity assessment and comparison of the land snail’s Cornu aspersum responses against CuO nanoparticles and ZnO nanoparticles. Comp. Biochem. Physiol. Part-C Toxicol. Pharmacol..

[142] Husen A., Iqbal M., Aref I.M. (2014). Growth, water status, and leaf characteristics of Brassica carinata under drought and rehydration conditions. Rev. Bras. Bot..

[143] El-Beltagi H., Mohamed H. (2013). Reactive Oxygen Species, Lipid Peroxidation and Antioxidative Defense Mechanism. Not. Bot. Horti Agrobot. Cluj-Napoca.

[144] Bisht G., Zaidi M.G.H., Sandeep A. (2014). Impact of Gold Nanoparticles on Physiological and Biochemical Characteristics of Brassica juncea. J. Plant Biochem. Physiol..

[145] Barbero F., Mayall C., Drobne D., Saiz-Poseu J., Bastús N.G., Puntes V. (2021). Formation and evolution of the nanoparticle environmental corona: The case of Au and humic acid. Sci. Total Environ..

