# Supplementary material for: Growth-Promoting Gold Nanoparticles Decrease Stress Responses in Arabidopsis Seedlings
Source: Nanomaterials (Basel). 2021 Nov 23;11(12):3161. doi: 10.3390/nano11123161 (PMC8707008; doi:10.3390/nano11123161)
Supplement: Supplementary file 1 [file nanomaterials-11-03161-s001.zip › Supplemental Figures corrected proofs.pdf]

Supplementary Materials

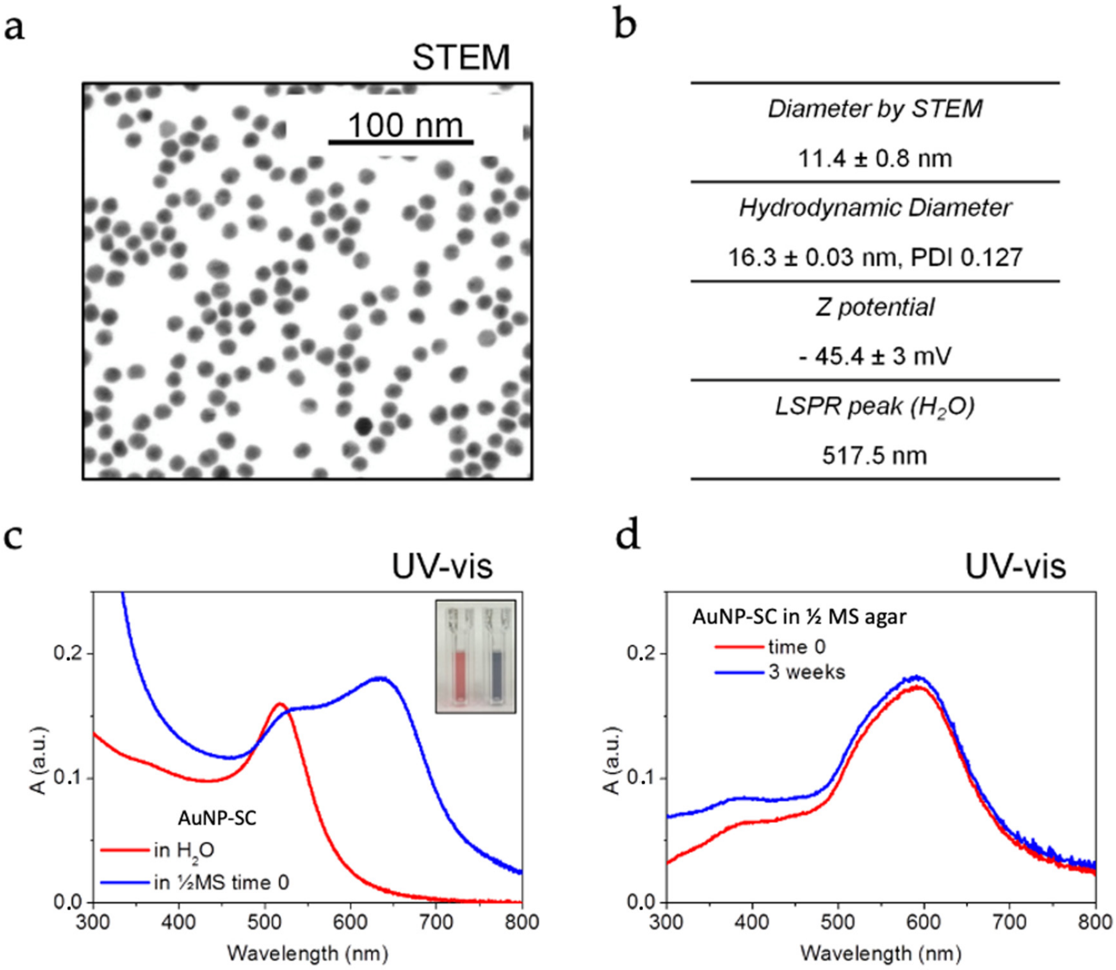

Figure S1: Physicochemical characterization of AuNP-SC dispersed in H_2O, ½ MS and ½ MS agar. **(a)** Bright field - scanning transmission electron microscopy (STEM) of AuNP-SC **(b)** NP average diameter measured by STEM; hydrodynamic diameters (in H_2O) measured by dynamic light scattering, reported as Z average and poly dispersity index (PDI); Z potential of the AuNPs dispersed in H_2O (pH 6.5, conductivity 0.77 mS/cm). **(c)** UV-vis spectra of AuNP-SC dispersed in H_2O (red) and in ½ MS at time 0 (blue); photograph of the AuNP-SC dispersed in H_2O (left) and in ½ MS (right). **(d)** UV-vis spectra of AuNP-SC dispersed in ½ MS agar at time 0 (red) and after 3 weeks of exposure (blue); a cuvette with unsupplemented solidified agar was used as reference. Absorbance A in arbitrary units (a.u.). All experiments were repeated two times with similar results.

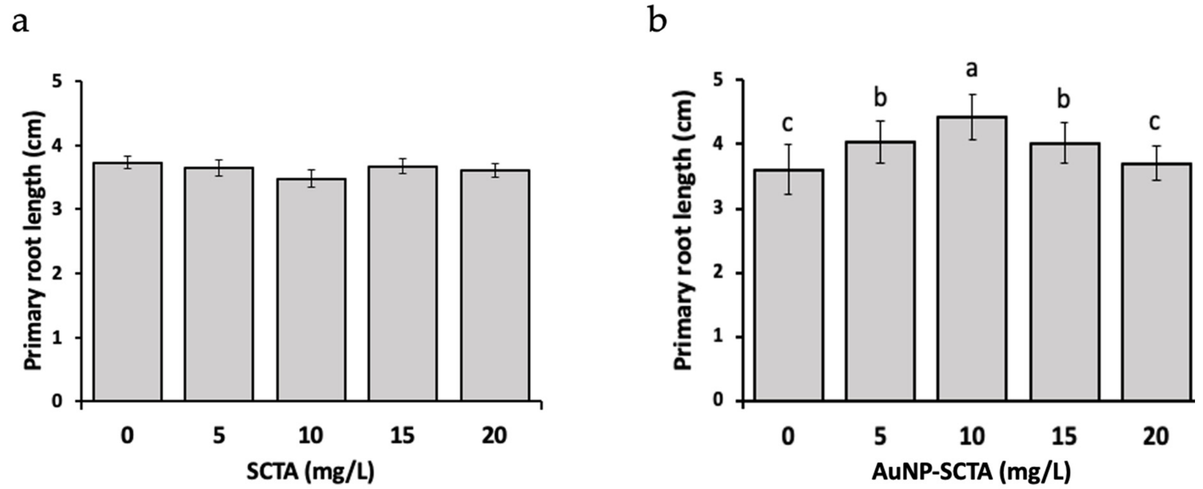

Figure S2: Growth of Arabidopsis seedlings in the absence and presence of AuNPs. **(a)** The NP stabilizer SCTA does not affect the primary root length at any of the tested concentrations, while **(b)** exposure to AuNP-SCTA promotes Arabidopsis root growth in dose-dependent mode. Wild-type Arabidopsis seedlings were grown for 7 d on agar-solidified ½ MS medium containing different concentrations of SCTA or AuNP-SCTA, in a concentration range from 0 to 20 mg/L. Primary root length was measured. Results shown are means ± SE (n=20). Different labels a-c indicate statistically different groups according to multiple comparisons following one-way ANOVA analysis at a probability level of $p < 0.01$. All experiments were repeated two times with similar results. The experiments were repeated two times with similar results.

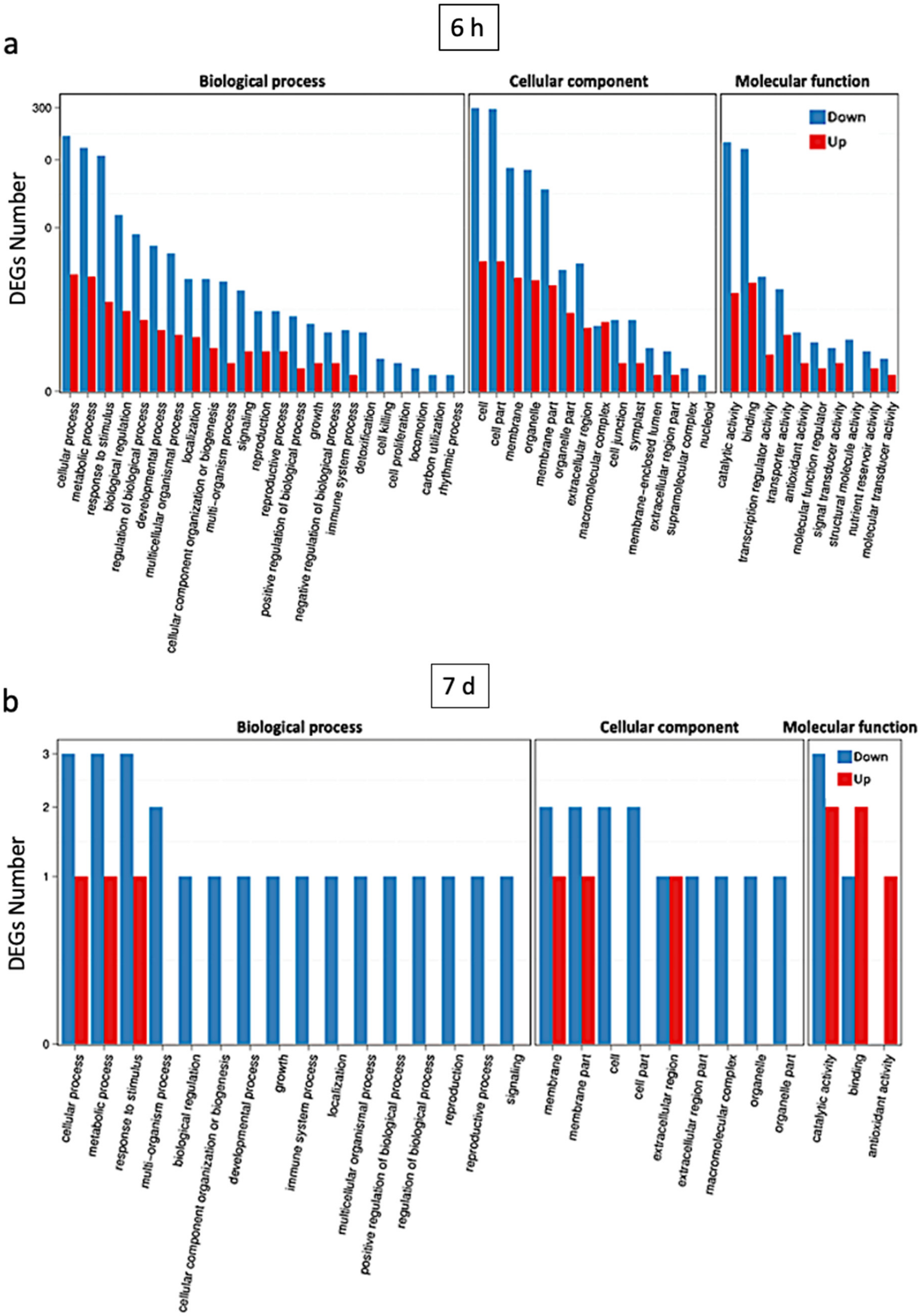

Figure S3: GO classification of DEGs after AuNP-SCTA treatment. Number of DEGs in the most enriched GO terms detected in Arabidopsis roots after **(a)** 6 h and **(b)** 7 d of AuNP-SCTA treatment. X axis represents GO term; Y axis represents the amount of up (red) and down (blue) regulated genes. DAVID database was used for the Gene Ontology functional annotation cluster analysis.

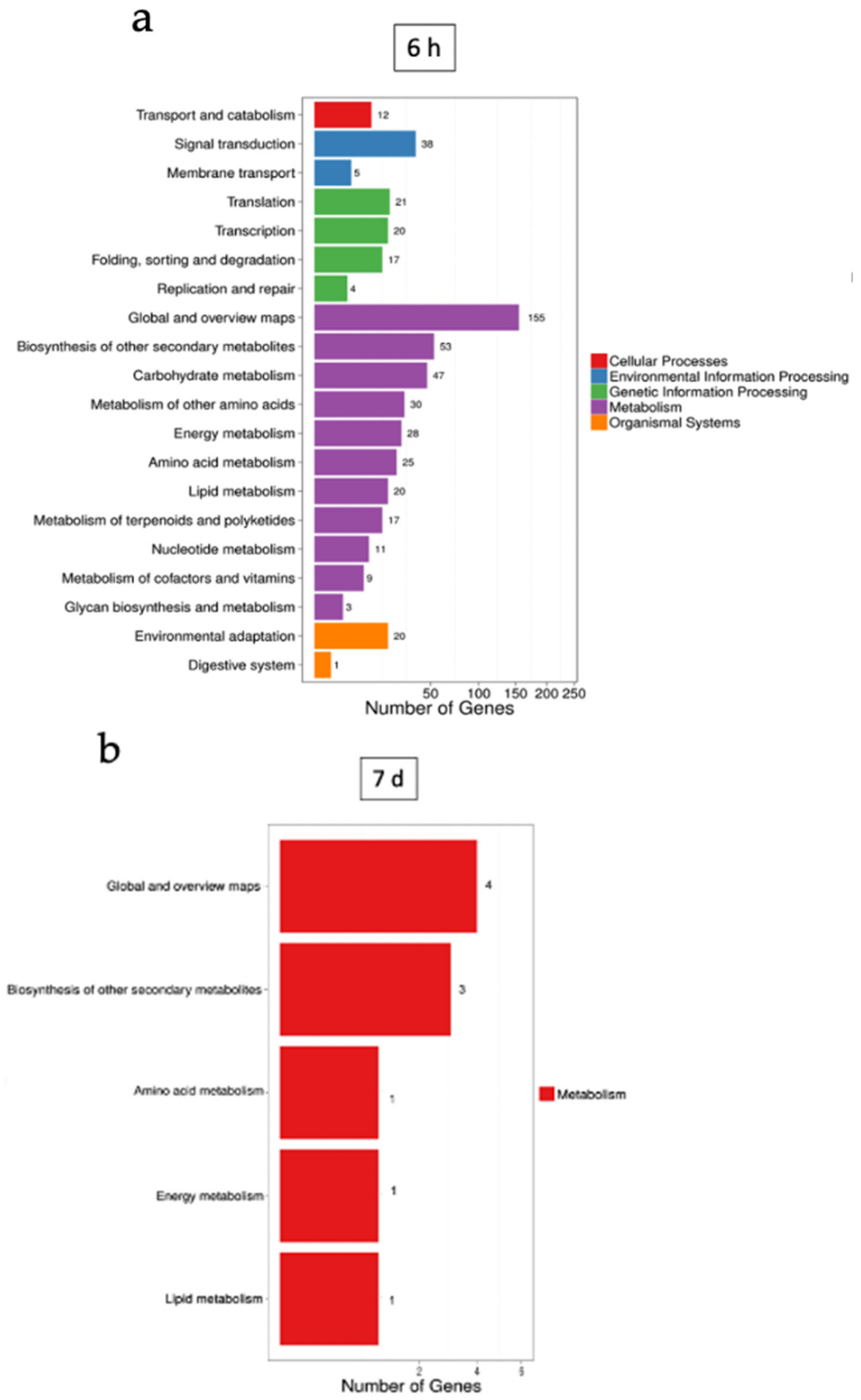

Figure S4: Pathway classification of DEGs after AuNP-SCTA treatment. Functional classification of DEGs identified in Arabidopsis roots after **(a)** 6 h and **(b)** 7 d of AuNP-SCTA treatment into KEGG (Kyoto Encyclopedia of Genes and Genomes) pathways. X axis represents number of DEGs; Y axis represents functional classification of KEGG.

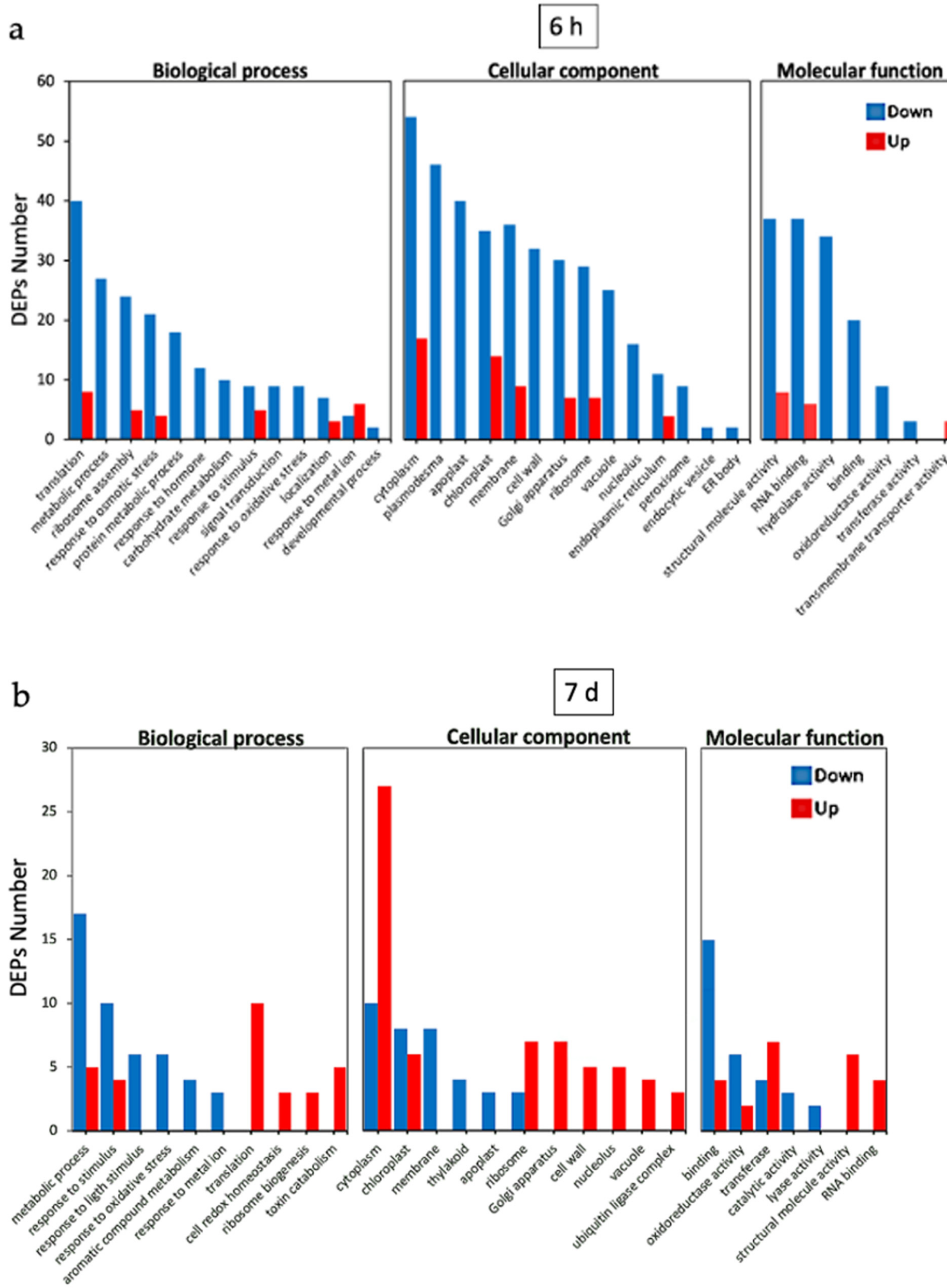

Figure S5: GO classification of DEPs after AuNP-SCTA treatment. Number of DEPs in the most enriched GO terms detected in Arabidopsis roots after **(a)** 6 h and **(b)** 7 d of AuNP-SCTA treatment. X axis represents GO term; Y axis represents the amount of up (red) and down (blue) regulated proteins. DAVID database was used for the Gene Ontology functional annotation cluster analysis.

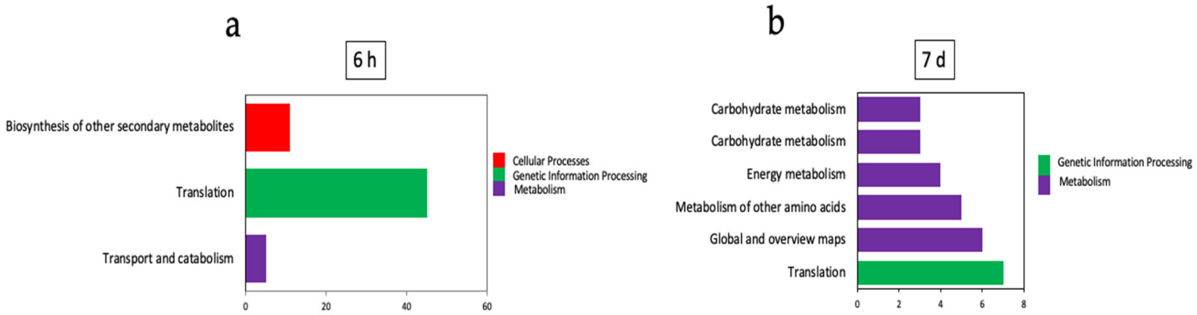

Figure S6: Pathway classification of DEPs after AuNP-SCTA treatment. Functional classification of DEPs identified in Arabidopsis roots after **(a)** 6 h and **(b)** 7 d of AuNP-SCTA treatment into KEGG (Kyoto Encyclopedia of Genes and Genomes) pathways. X axis represents number of DEGs; Y axis represents functional classification of KEGG.

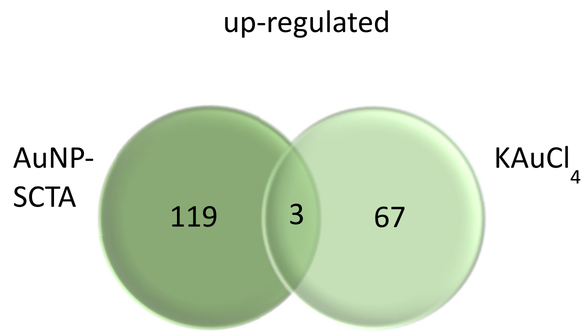

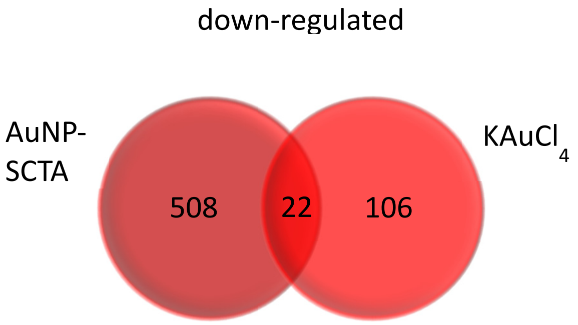

Table of co-upregulated genes

AT1G07610	METALLOTHIONEIN 1C (MT1C)
AT1G08430	ALUMINUM-ACTIVATED MALATE TRANSPORTER 1 (ALMT1)
AT5G25460	DUF642 L-GALL RESPONSIVE GENE 2 (DGR2)

Table of co-downregulated genes

AT1G02920	GLUTATHIONE S-TRANSFERASE 7 (GSTF7)
AT1G02930	GLUTATHIONE S-TRANSFERASE 6 (GSTF6)
AT1G05880	ARIADNE 12 (ARI12)
AT1G26240	EXTENSIN 19 (EXT19)
AT1G26380	FAD-LINKED OXIDOREDUCTASE 1 (FOX1)
AT1G26410	FAD-binding Berberine family protein(ATBBE6)
AT1G33900	IMMUNE ASSOCIATED NUCLEOTIDE BINDING 4 (IAN4)
AT1G67980	CAFFEOYL-COA 3-O-METHYLTRANSFERASE (CCOAMT)
AT2G02930	GLUTATHIONE S-TRANSFERASE F3 (GSTF3)
AT2G26560	PHOSPHOLIPASE A 2A (PLA2A)
AT2G30660	ATP-dependent caseinolytic protease/crotonase family protein
AT2G30670	NAD(P)-binding Rossmann-fold superfamily protein
AT2G30750	CYTOCHROME P450, FAMILY 71, CYP71A12
AT2G39400	(MAGL6)
AT2G43510	TRYPSIN INHIBITOR PROTEIN 1 (TI1)
AT2G43570	CHITINASE, PUTATIVE (CHI)
AT4G32950	protein phosphatase 2v family protein
AT5G02780	GLUTATHIONE TRANSFERASE LAMBDA 1 (GSTL1)
AT5G13320	AVRPPHB SUSCEPTIBLE 3 (PBS3)
AT5G19890	peroxidase family protein
AT5G39120	RmlC-like cupins superfamily protein
AT5G39150	RmlC-like cupins superfamily protein

Figure S7: Venn diagram of DEGs after AuNP-SCTA exposure (this study) and DEG after exposure to $KAuCl_4$ resulting in *in planta* AuNP formation [140]. Table of the overlap between our DEGs and those published by Tiwari et al. (2016).
